# Trait Mindfulness Supports Self-perceived Scholastic Competence in Adolescent Girls

**DOI:** 10.1525/collabra.57559

**Published:** 2023-01-13

**Authors:** Clare F. McCann, Theresa W. Cheng, Arian Mobasser, Jennifer H. Pfeifer, Kathryn L. Mills

**Affiliations:** 1 Psychology, University of Oregon, Eugene, OR, US; 2 Psychology, University of California, Los Angeles, CA; 3 Psychiatric and Neurodevelopmental Genetics Unit, Massachusetts General Hospital, Boston, MA, US; 4 Psychology, PROMENTA Research Center, University of Oslo, Oslo, Norway

**Keywords:** education, longitudinal, middle school, identity development

## Abstract

Identity development is a core task of adolescence. Self-perceptions of scholastic competence are tied to the academic domain of identity development and have immediate consequences for educational attainment. Understanding the malleability of self-perceptions of scholastic competence, and the factors which may influence its developmental course, are crucial for efforts to improve educational outcomes. This preregistered longitudinal study describes how self-perceived scholastic competence changes across early adolescence, relates to trait mindfulness, and is impacted by school transitions. We investigated these questions in 174 adolescent girls (10–16 years), who each contributed up to three waves of data, using multilevel modeling. Our results demonstrated that prior levels of self-reported mindfulness and school transitions are positively related to self-perceived scholastic competence, whereas age is not.

Scholastic competence is an important domain of adolescent identity development because it has been found to promote academic achievement (e.g., Guay et al., 2003; Marsh et al., 2005; Marsh & Martin, 2011) and occupational aspirations (Ireson & Hallam, 2009). However, adolescents can also develop more negative views about their academic performance and about themselves generally (e.g., Baldwin & Hoffmann, 2002; Eccles et al., 1984; Onetti et al., 2019). Since academic self-concept is tied to academic achievement, it is important to identify the factors relating to the development of this facet of identity.

There are contradicting findings related to developmental patterns in self-perceptions. In the past, it has been suggested that academic self-perceptions are relatively less differentiated in childhood, and then it becomes increasingly differentiated (including less global positivity) in adolescence (Shavelson et al., 1976; Wiley, 1979). However, individual trajectories might deviate from previously identified group-level trajectories (Białecka-Pikul et al., 2019). By characterizing developmental changes in self-perceived scholastic competence in adolescence, we can better understand how this aspect of self-concept changes on average, as well as how variable this change is across individuals.

Both individual-level factors and environmental-factors can have an impact on the development of perceived scholastic competence. For instance, previous research done with both adolescents and young adults suggests that with higher levels of trait mindfulness—defined as the ability to reflect on one’s own current state—are more likely to demonstrate positive global self-worth and self-concept (Brown & Ryan, 2003; Greco et al., 2011). Environmental factors such as transitioning to a new school have been found to increase the level of instability in self-concept development in adolescence (e.g., Białecka-Pikul et al., 2019; Cole et al., 2001). The current study investigates these two factors in a longitudinal study to specify how transitions into middle school, and levels of trait mindfulness, relate to the self-perception of scholastic competence in adolescent girls,^1^ who have been found to be at increased risk for more negative self-concepts (Coelho & Romão, 2017).

## School Transition & Self-Perceptions

Transitioning to a new school can be intimidating, as it brings new experiences, new people, and flux in self-perceptions at a time of profound individual identity development. In general, there is an increase in social comparison among peers or a more intense self-evaluation that stems from the transition into adolescence (Cole et al., 2001; Feldlaufer et al., 1988). School transitions can promote instability in self-perception development in adolescence (e.g., Białecka-Pikul et al., 2019; Cole et al., 2001). In previous longitudinal studies, it has been found that children and younger adolescents experience a significant decrease in self-perceived academic competence after the transition from elementary to middle school over and above age at the time of self-report (typically after grades 5–7; e.g., Cantin & Boivin, 2004; Coelho & Romão, 2017; Cole et al., 2001; similar findings have come from cross-sectional studies such as Onetti et al., 2019). Increased negativity about the self in academic domains might be attributed to new grading practices, teaching expectations, student-teacher relationships, and educational settings (e.g., Cantin & Boivin, 2004; Eccles et al., 1993; von Keyserlingk et al., 2019). In the United States educational system, studies have found that middle schoolers report, on average, more negative academic self-concept than high schoolers (e.g., Cole et al., 2001; Murdock et al., 2000). This suggests that the transition to high school might be associated with changes that may result in increased positivity of scholastic self-perceptions. The work summarized here tells us that we know shifts in self-perception of scholastic competence occur with school transitions, but the directionality of this instability needs further examination.

We focus on girls because a study in Portugal found that girls experience a significant decrease in positive academic self-concept following the transition to secondary school (Coelho & Romão, 2017). Furthermore, the age range of 10–14 years brings the start of puberty. Puberty is accompanied by a host of transitions including shifts in values, motivation, and goals related to identity (Pfeifer & Allen, 2021; Pfeifer & Berkman, 2018). Individuals who have had their first period could be more vulnerable to the negative effect of a school transition (Cantin & Boivin, 2004; Juraska & Willing, 2017; Simmons et al., 1987). Specifically, Cantin & Boivin (2004) found that individuals who experienced menarche prior to the transition into middle school had higher rates of negative self-perceptions, over individuals who had not experienced menarche. Therefore, further understanding how academic self-perceptions changes over time in girls is important because they may have biological and social vulnerabilities to poorer self-concept during social transitions. Further, distinguishing the impact of the transition into middle school as compared to the transition into high school on self-perceived scholastic competence is needed to understand the unique challenges of transitions in hopes to promote more positive self-perceived scholastic competence during the early adolescent and mid-adolescent period.

## Mindfulness & Self-Perceptions in an Academic Context

As described previously, one individual-level factor that might be useful in promoting scholastic self-competence is mindfulness. Mindfulness is commonly defined as non-evaluative, non-elaborative attention and awareness of an individual’s own current experience (Brown & Ryan, 2003). The concept of mindfulness stems from a Buddhist tradition; however, it is not always considered to be a religious trait (Rayan & Ahmad, 2016). The present study will be examining *trait* mindfulness, which is commonly assessed by the degree to which individuals observe internal experiences, act with awareness, and accept internal experiences without judging them (Baer et al., 2006), rather than *state* mindfulness which is practiced in mindfulness meditation (e.g., Lau et al., 2006). Trait mindfulness is also different than *metacognition*, which is defined as knowledge or beliefs about thinking and strategies used to regulate and control thinking processes (Flavell, 1979). In the present study, we examine trait mindfulness, which can be increased with training, and its relationship to self-perceived scholastic competence.

Among adolescents, increasing levels of mindfulness are related to higher academic functioning (Greco et al., 2011) and stronger global self-worth (Brown & Ryan, 2003). Expressing and implementing mindfulness in the school-based context is recognized by educators as being associated with fostering attention, resilience, and well-being (*Garrison Institute, 2005*). These findings, among others discussed below (see Carlson, 2013), suggest that mindfulness is a potentially malleable factor related to academic achievement, self-perceptions, and well-being. Due to the malleable nature of trait mindfulness, we propose gaining further understanding of the relationship between trait mindfulness and self-perceived scholastic competence could have implications in improving how individuals feel about themselves in an academic setting. For example, if higher levels of trait mindfulness is found to be related to increased self-perceived scholastic competence than future work could focus on improving trait mindfulness to mimic this effect.

Individuals with higher levels of trait mindfulness are better able to overcome barriers that often impede accurate general self-perceptions (Brown & Ryan, 2003; Carlson, 2013). A theoretical framework proposed by Bishop and colleagues (2004) suggests that mindfulness leads to clearer self-perceptions by allowing individuals to pay more attention to their current experiences within themselves. Trait mindfulness reduces reactivity and defensiveness when confronted with ego-threatening information which should in turn reduce bias when processing information about one’s personality (Carlson, 2013). These findings suggest that mindfulness, both trait-level and practiced, is a potential malleable factor relating to academic achievement, perception, and well-being.

To help lay a foundation for understanding how mindfulness and self-perceived scholastic competence relate across adolescence, we need to assess whether trait mindfulness is a malleable factor promoting positive academic self-perception in a longitudinal study. By doing so, we can possibly increase the motivation to implement mindfulness learning.

## Pre-registered Objectives and Hypotheses

The present study examines both trait mindfulness and school transitions as separate potential mechanisms contributing to the instability in self-perceptions across ado lescence, and later on in a post-hoc analysis, examines them together.

The first objective of this study is to examine how self-perceived scholastic competence changes across time in adolescent girls. Due to prior literature reviewed above, we hypothesize that self-perceived scholastic competence will increase on average suggesting that the increase from middle school to high school surpasses the original decrease following elementary school.

The second objective of this study is to examine how levels of self-perceived scholastic competence are impacted by the transition into middle school and high school. Given prior findings as discussed above, we hypothesize that the transition to middle school will predict, on average, a decrease in self-perceived scholastic competence while the transition into high school will predict, on average, an increase in self-perceived scholastic competence.

The final objective of this study is to understand if mindfulness predicts self-perceived scholastic competence. We hypothesize that higher prior levels of mindfulness will predict higher levels of scholastic competence, as higher levels of mindfulness have been shown to relate to higher academic functioning and general self-perceptions (Carlson, 2013; Greco et al., 2011).

## Methods

### Participants.

174 female^2^ adolescents aged 10.0 to 13.0 years were recruited through letters distributed by schools in the Lane County, Oregon area, recruitment flyers, and secure databases. Recruitment targeted 5th and 6th graders who were registered as female by the school. Originally recruited 189 participants but 15 individuals were excluded based on predetermined exclusionary criteria: diagnosed with a developmental disability, psychotic disorder, behavioral disorder; taking psychotropic medication other than stimulants, MRI contradictions, report or suspect being pregnant. For each participant, we collected up to three waves of data spaced approximately 18–24 months apart. Refer to Table 1 for the characteristics of the sample. This information was reported by parents on a demographic questionnaire and by the participant during a diagnostic interview and was further cross-checked when there were discrepancies. All participants and parents provided written informed consent (parental consent and participant assent) at each session. Participants were compensated through cash or check at the end of each session. Ethics approval was received by the Institutional Review Board of the University of Oregon.

### School Characteristics.

At wave 1 (n=174): 92% of participants attended public schools, 2% of participants attended private school, 2% of our participants were homeschooled and 4% were in either Montessori, charter schools, or immersion programs. At wave 2 (n=163): 91% of participants attended public schools, 2% of participants attended private school, 1% of participants attended did not attend school, 1% of participants attend the cyber or online school, 2% of participants were homeschooled and 2% of participants Montessori, charter schools or immersion programs. At wave 3, prior to COVID-19 (n=101): 90% of participants attended public school, 4% of participants attended private school, 3% of participants attended cyber or online school, 2% of participants were homeschooled and 1% of participants attended an art school.

### Transition characteristics.

In wave 1 (n=174), 41% of participants were in elementary school and 58% of participants were in middle school. In wave 2 (n=163), 96% of participants were in middle school and 4% were in high school. In wave 3 (n=93), 23% of participants were in middle school and 77% were in high school.

### Data Collection.

The data for this project is from the Transitions in Adolescent Girls (TAG) study at the University of Oregon funded by the National Institutes of Mental Health. TAG is a longitudinal study that aims to understand relationships between pubertal development, brain structure and connectivity, the behavioral and neural cor relates of social and self-perception processes, and adolescent mental health in girls. This study used data accumulated from three waves over the course of four years. There are two sessions within each wave, spaced approximately 4 weeks apart. The first session is a diagnostic interview, and the second session consists of functional magnetic resonance imaging scans. In between those two sessions, the participants complete saliva samples to assess hormone function and three sets of questionnaires. Data collection from wave one (n=174) and wave two (n=163) is complete. The sample size at the third wave (N=101) is not true attrition due to individuals dropping out, it was based on the schedule of initial recruitment. We used data collected up until January 13th, 2020 (pre-COVID scare and lockdown) for simplicity and to meet necessary timelines for the first author’s degree requirements. Data collection was paused for the TAG study for 8–9 months, after which it resumed and approximately 60 more participants completed a third wave of data collection. The degree of attrition over each wave in the study has averaged ~ 5%. Regarding bias in the sample, we have observed that the age at initial recruitment tended to decrease over the duration of the first wave (participants in older grade levels were recruited first until we expanded to include a younger grade level, even though the age range for initial inclusion did not change). Therefore, it is likely that some of the very youngest participants in the study overall provided data for the third wave *after* the pandemic, and those are the participants whose data are missing at the third wave. Finally, using data collected after the pandemic, given the extensive disruptions to schooling and in-person education, is interesting but complex for those reasons; therefore, it best represents a promising future direction for additional research.

**Figure 1.**
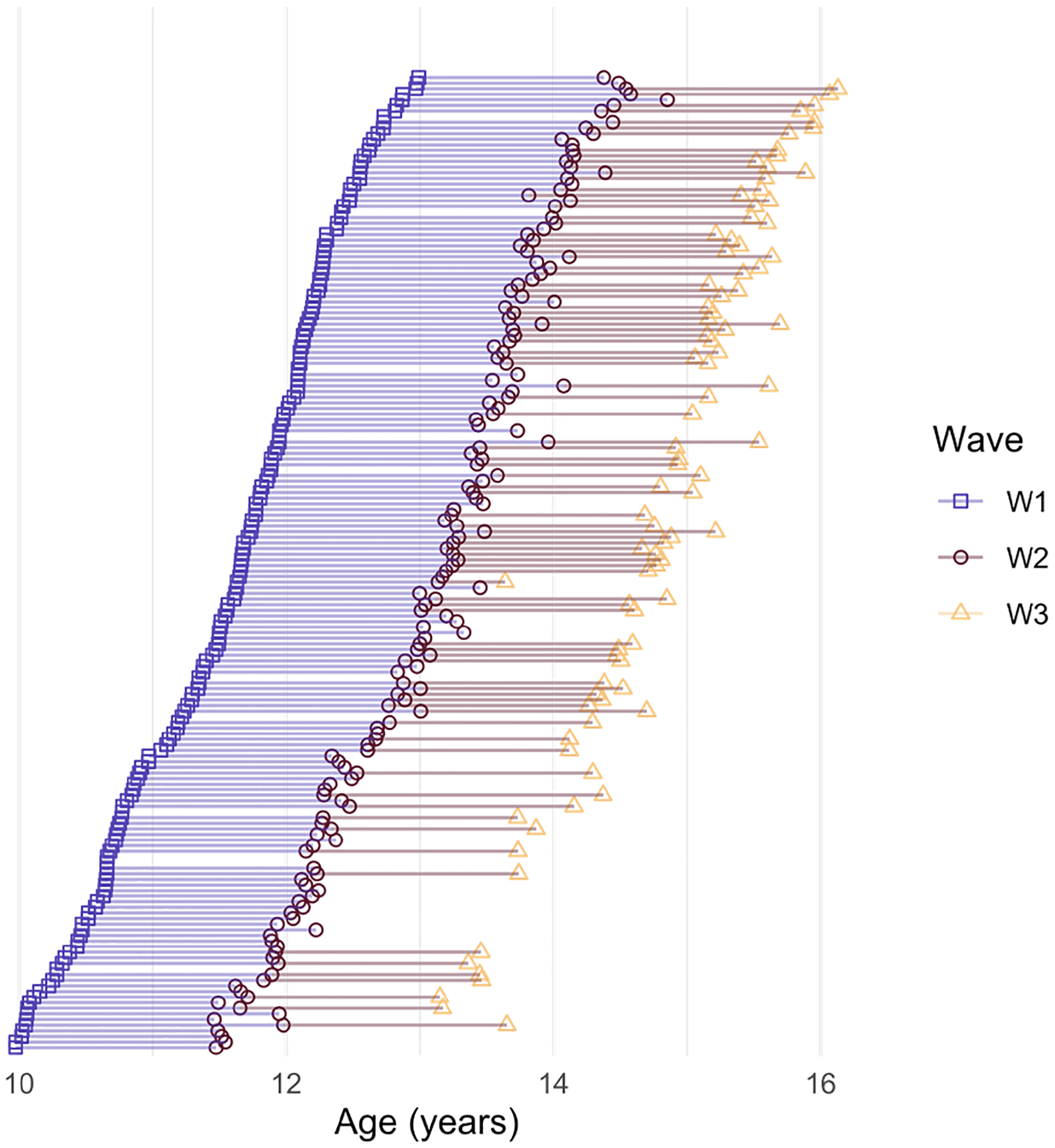
Study design plot of participation across the three waves of data used in the present study. Each line represents one participant, each dot linking the lines represents one data point contributed. Purple squares indicate wave one, magenta circles indicate wave two, and yellow triangles indicate wave three. The x-axis is each participant’s age during the corresponding wave of participation.

For more information on the TAG protocol see Barendse et al. (2020). See Figure 1 for a visualization of data collection across the three-waves up until January 13th, 2020.

## Measures

### Self-Perceived Scholastic Competence.

Self-perceived scholastic competence was operationalized as the Scholastic Competence (SC) subscale from the well-validated Self-Perception Profile for Adolescents (SPPA; Harter, 2012), which the participants completed in its entirety. This subscale includes items that refer to the individuals’ perceived cognitive competence, as applied to schoolwork. When filling out the questionnaire, participants see two statements and first decide which statement is more like them. An example of an item is “Some teenagers feel like they are just as smart as others their age BUT Other teenagers aren’t so sure and wonder if they are as smart”. Next, they rate how much the statement is like them, selecting from the options “Sort of True” or “Really True”. The subscale is made up of five items, keyed as either *positive* (+), meaning that the more positive competent self-description is the first statement seen by the participant, or *negative* (−), meaning the less competent self-description is presented as the first statement seen by the participant. Negative items are reverse scored. Cronbach’s alpha for the first wave is 0.78, the second wave is 0.80 and the third wave is 0.72. Each participant’s SPPA-SC score was calculated as the sum across items (range = 5–20). Higher scores indicate a higher perception of scholastic competence. One participant from wave one was excluded from analyses for missing more than one of the five items (20%) from the SPPA-SC subscale.

### Mindfulness.

We used the well-validated Child and Adolescent Mindfulness Measure (CAMM; Greco et al., 2011; Kuby et al., 2015), a survey that identifies levels of trait mindfulness by assessing the degree to which children and adolescents observe internal experiences, act with awareness, and accept internal experiences without judging them. In this survey, participants are asked to rate a total of 10 items on a 5-point Likert scale of “Never True”, “Rarely True”, “Sometimes True”, “Often True” and “Always True”. Examples of items from the scale are “I get upset with myself for having feelings that don’t make sense” and “At school, I walk from class to class without noticing what I’m doing”. Cronbach’s alpha is 0.88 for both the first and second wave and is 0.91 for the third wave. Each participant’s CAMM score was calculated as the sum across items (range = 0–40). Higher scores indicated the increased tendency to express trait mindfulness in everyday life. Two participants at wave one and wave two were missing 20% or more of the items and were thus excluded from further analyses.

### School Transition.

Participants were asked what grade they were currently in at each wave using an interview and questionnaire. Transitioning was defined as moving from elementary to middle school or middle school to high school, indicated as the move from grades five to six or grade eight to nine, respectively. If this move occurred between waves, it was coded as a transition. For example, if a participant completed wave 2 in January of 2018, moved to middle school in September of 2018 and then came in for their wave 3 in March of 2019 they would be considered someone who experienced a transition in between waves. At W1, 92 participants had transitioned into middle school from elementary school. From W1-W2: 69 participants transitioned to middle school, 5 participants transitioned to high school and 89 participants did not transition. From W2-W3: 70 participants transitioned from middle to high school and 23 participants did not transition. Data was coded at each wave to indicate whether the participant had transitioned to middle school or high school since the last assessment (0 = No Transition, 1 = Middle School, 2 = High School).

### Household Income Bracket.

Household income bracket was used as a categorical covariate due to findings from Eshel and Klein (1981), revealing that children from low-income families have lower levels of accuracy regarding academic self-concept in early years of schooling, in comparison to children from middle-class families. Parental self-report of the family income bracket at wave one was the pre-registered variable was used as a proxy for SES. The question asked was “What is the general level of your annual total household income?”. Household income bracket was coded as follows: 1 = up to 25,000; 2 = 25,000 to 40,000; 3 = 40,000 to 75,000; 4 = 75,000 to 100,000; 5 = over 100,000; 6 = don’t know; 7 = decline to respond. We further simplified these income brackets into three separate categories using median household income from Lane County, Oregon reported by Data USA (https://datausa.io/profile/geo/lanecounty-or). Those who reported making less than 40,000 a year are labeled “low income,” those who reported making 40,000 to 75,000 a year are labeled “middle income,” those who reported making over 75,000 are labeled “high income.”

### Age at Menarche.

We used the participant’s self-reported age at menarche as a covariate in our analysis due to findings discussed in the introduction.

### Pre-registered Statistical Analyses

The goals of this study were to understand how self-perceived scholastic competence changes across adolescence in girls, how levels of self-perceived scholastic competence are predicted by the transition into middle school and high school, and how much levels of self-perceived scholastic competence are predicted by prior levels of mindfulness. The dependent variable in all the models was self-perceived scholastic competence. We applied pre-registered multilevel models (see model breakdown below) to test the questions described above using the *R* package *nlme* (Pinheiro et al., 2007). The data were clustered at the participant level. Age at menarche and household income bracket were both used as covariates in all analyses. All coefficients reported are unstandardized and variables will be examined simultaneously. The pre-registration for the present study can be found at https://osf.io/uynpm/. See Table 2 for information regarding missingness.

Akaike Information Criterion (AIC) values were compared between the null model and the model including predictors to assess which one was most parsimonious (Akaike, 1974) as this is a standard operating procedure in our lab. We also assessed the likelihood ratio statistic to determine if a more complex model was significantly different from the simpler model in explaining the relationship between self-perceived scholastic competence and the following predictors for each question, (1) age, (2) prior levels of mindfulness, and (3) school transitions.

Here is an example of a multilevel model: *SPPA-SC*_*ij*_ = *B*_*0j*_ + *B*_*1j*_*Mindfulness*_*ij*_ + *e*_*ij*_. *SPPA-SC*_*ij*_ represents the dependent variable which is self-perceived scholastic competence for the present study. The “*i*” subscript represents the score at a certain level or time point, and the “*j*” subscript represents the group which is each participant for the present study. Mindfulness_ij_ represents the level 1 predictor, *B*_*0j*_ is the intercept of the dependent variable in group “*j*” or level 2, *B*_*1j*_ refers to the slope for the relationship in group “*j*” or level 2 between the level 1 predictors and the dependent variable. *e*_*ij*_ represents the random errors of prediction for the level 1 equation

#### Self-Perceived Scholastic Competence and Age.

The first aim of the study was to understand whether self-perceived scholastic competence changed across early adolescence. We tested to see if self-perceived scholastic competence changed with age in our sample by comparing models including linear and quadratic terms to a null model, including household income bracket and age at menarche as covariates. We tested each model against a null model with just the covariates as fixed effects and a random intercept for each participant. Our age models included polynomial terms (linear and quadratic) for mean-centered age.

#### Self-Perceived Scholastic Competence & School Transition.

The second aim of this study was to understand how levels of self-perceived scholastic competence are predicted by the transition into middle school and high school. We compared a model including school transitions as the predictor of self-perceived scholastic competence while controlling for prior self-perceived scholastic competence to a null model using only a random intercept, age at menarche, and SES for each participant.

#### Self-Perceived Scholastic Competence & Mindfulness.

The third aim of this study was to understand how prior levels of trait mindfulness predict self-perceived scholastic competence. We compared a model including prior levels of mindfulness as the predictor of self-perceived scholastic competence while controlling for prior levels of self-perceived scholastic competence to a null model using only a random intercept, age at menarche, and SES.

## Results

### Self-Perceived Scholastic Competence and Age.

We did not observe a linear or quadratic relationship between age and self-perceived scholastic competence at the group-level.

### Self-Perceived Scholastic Competence & School Transition.

Our model including prior levels of self-perceived scholastic competence, controlling for household income bracket, prior levels of self-perceived scholastic competence, and age at menarche explained significant variance in self-perceived scholastic competence following both the transition from middle school and high school. Likelihood ratio tests comparing the full model to one excluding school transitions and not controlling for prior levels of self-perceived scholastic competence show that the full model significantly improved the model without overfitting (χ2(2) =8.63, *p*<0.001, AIC difference=5; Table 3). These results suggest that with the transition into middle school and the transition into high school, the self-perceived scholastic competence score increases on average.

### Self-Perceived Scholastic Competence & Mindfulness.

**Figure 3.**
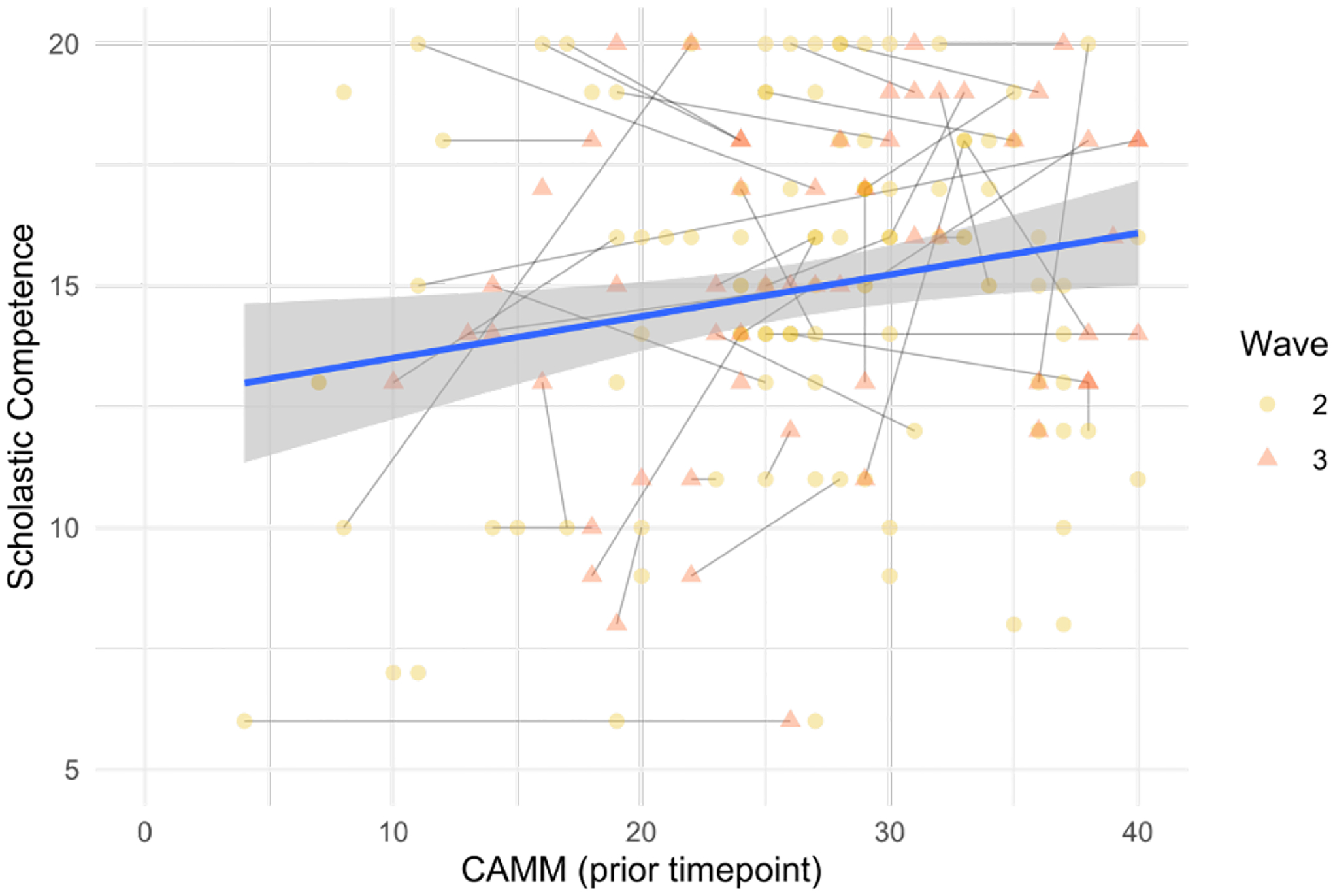
Graph of Self-Perceived Scholastic Competence and Mindfulness at the prior wave. CAMM, on the X-axis, is the Child and Adolescent Mindfulness Measure. Each dot represents a data point and linked data points represent a single participant. Mustard yellow triangle indicates wave 2 self-perceived scholastic competence scores and salmon-colored square indicate wave 3 self-perceived scholastic competence scores. Light blue circles would indicate wave one self-perceived scholastic competence scores but are not included as later waves of scholastic competence scores were outcome variables.

Our model including prior levels of mindfulness, controlling for prior levels of self-perceived scholastic competence, household income bracket, and age at menarche, explained significant variance in subsequent self-perceived scholastic competence. Likelihood ratio tests comparing the full model to one excluding prior mindfulness scores as a pre dictor shows that including prior mindfulness scores significantly improved the model without overfitting (χ2(1) =8.20, *p*=0.004, AIC difference=6; Table 4). Those who displayed higher prior levels of trait mindfulness also showed higher levels of self-perceived scholastic competence. Figure 3 depicts the relationship between prior levels of mindfulness and self-perceived scholastic competence.

## Deviations from Pre-Registration

We conducted additional post-hoc analyses that deviated from our pre-registered analysis plan. The nature of these post-hoc analyses was (a) to interrogate how including or not including time-lagged and contemporaneous versions of dependent (self-perceived scholastic competence) and independent (levels of mindfulness) variables impacted our findings, and (b) to investigate more objective measures of scholastic competence (academic achievement).

### Are contemporaneous levels of mindfulness and self-perceived scholastic competence related?

We examined the relationship between contemporaneous measurements of self-reported mindfulness and self-perceived scholastic competence to expand our understanding of how these two processes are related outside of the lagged relationship and found that they are significantly related. These results demonstrate that individuals who self-report higher trait mindfulness are also more likely to concurrently report high levels of self-perceived scholastic competence (χ2(1) =9.92, *p*=0.002; Table 5).

### Academic Achievement.

During a diagnostic interview completed at the first session of each wave, participants are asked what their best, worst, and average letter grade was of the past year. We use the average grade reported at each wave (1=A, 2=B, 3=C, 4=Not passing).

### Analyses.

We wanted to know if our findings regarding self-perceived scholastic competence could be accounted for by actual academic achievement (i.e., letter grades), as prior work has identified a positive relationship between these variables (Guay et al., 2003; Marsh et al., 2005; Marsh & Martin, 2011). To do so, we conducted an analysis examining the relationship between self-perceived scholastic competence and average academic achievement. Likelihood ratio tests comparing the model including average academic achievement as a predictor of self-perceived scholastic competence to a null model show that including academic achievement significantly improved the model without overfitting (χ2(3) =27.71, *p*<0.001, AIC difference=22). Those who reported an average letter grade below an A had significantly lower self-perceived scholastic competence than those who reported having an average letter grade of A.

We conducted sensitivity analyses including academic achievement as an additional covariate in main models. With the inclusion of self-reported average letter grades, both the linear and quadratic relationships between age and self-perceived scholastic competence were still non-significant. Prior trait mindfulness also still explained variance in self-perceived scholastic competence after controlling for average letter grades (χ2(1) =15.32, *p*<0.001, AIC difference=13; Table 6).

We compared a model assessing the relationship between self-perceived scholastic competence and school transitions while controlling for average letter grades, household income bracket, and age at menarche to a model excluding school transitions as a predictor. We found that including school transitions still improved the model without overfitting (χ2(2) =7.20, *p*=0.027, AIC difference=3; Table 7). However, the transition into middle school no longer significantly explained variance in self-perceived scholastic competence.

**Figure 4.**
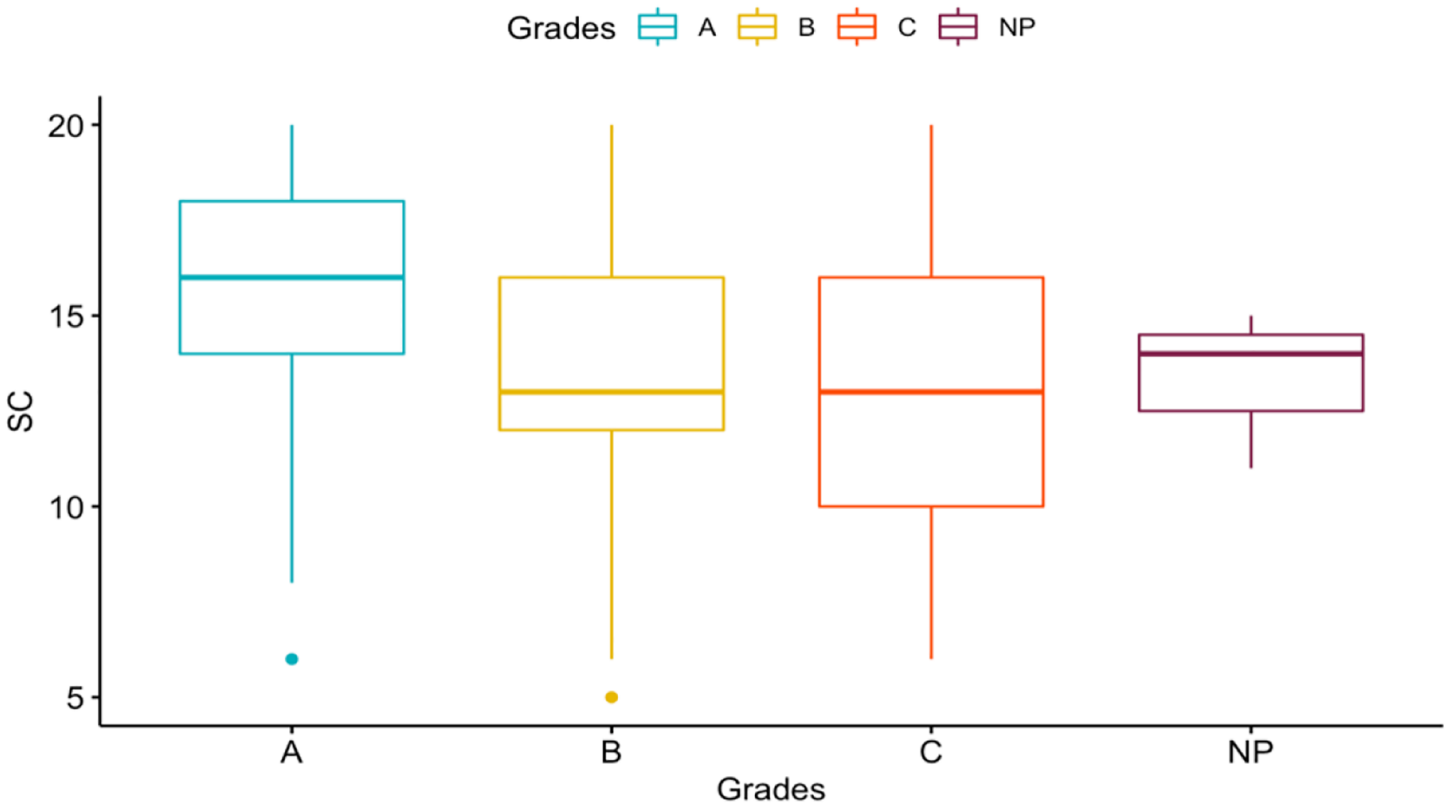
Boxplot of Self-Perceived Scholastic Competence and Average Letter Grades. The y-axis represents the self-perceived scholastic competence score, and the x-axis represents the average letter grade as reported by the participant. A is indicated with the light blue line and box, B is indicated by the yellow line and box, C is indicated as the orange line and box, Not Passing is indicated by the purple line and box.

Due to the previously mentioned literature suggesting a positive relationship between academic achievement and self-perceived scholastic competence, we wanted to see whether self-perceived scholastic competence predicts academic achievement. In cumulative link mixed models, both prior (*z*=−4.47, *p*<0.001, *OR*=.737 (95% CI: 0.645, 0.843)) and contemporaneous (*z*=−5.19, *p*<0.001, *OR*=.775 (95% CI: 0.704, 0.853)) levels of self-perceived scholastic competence significantly predict average letter grade. See Figure 4 for depiction of the relationship between self-perceived scholastic competence and academic achievement.

Previous literature suggests puberty may play a role in female adolescent’s self-perception trajectories during the transition from elementary to middle and middle to high school. To account for this, we ran a post-hoc analysis examining age at menarche as a moderator of the relationship between school transitions and self-perceived scholastic competence. We found that the addition of the age at menarche to the full model including SES, school transitions, and prior levels of self-perceived scholastic competence did not improve the model fit and the effect was not significant.

**Figure 5.**
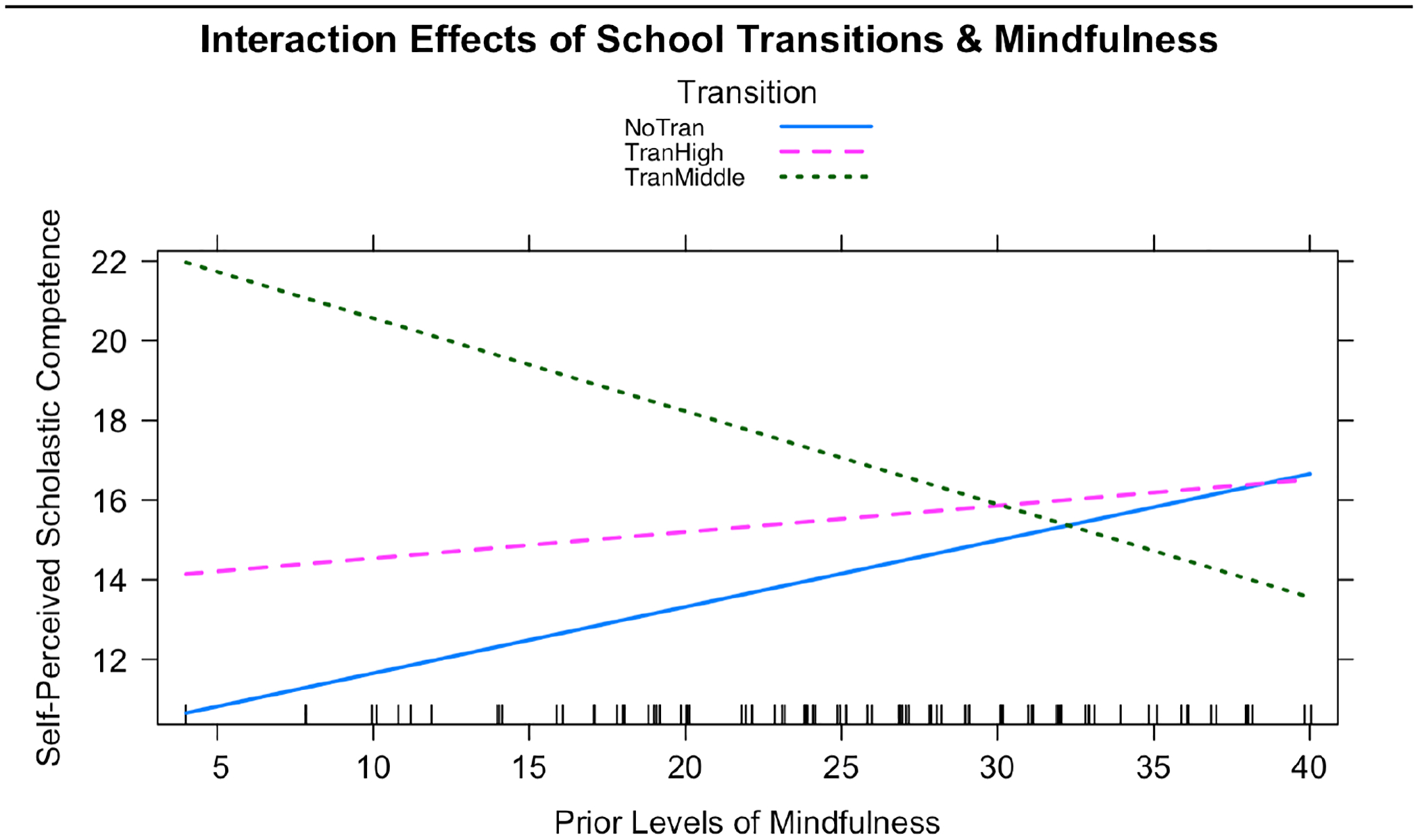
Plot of Simple Slopes from a Model Including an Interaction Between School Transitions and Mindfulness. The y-axis is the self-perceived scholastic competence score, and the x-axis is the prior level of mindfulness. The blue continuous line represents the simple slope for those that do not transition, the pink long dashed line represents the simple slope for those that transitioned into high school, and the green dotted line represents the simple slope for those that transitioned into middle school.

Finally, we wanted to explore the potential relationship between school transitions, mindfulness, and self-perceived scholastic competence all together. As both mindfulness and school transitions are significant predictors of self-perceived scholastic competence, we examined their interaction as a potential moderator of these relationships. We found that the inclusion of the interaction between prior levels of mindfulness and school transitions significantly improved model fit when added to a model including our covariates, prior levels of mindfulness, school transitions and a random intercept (χ2(12) =10.82, p=0.005, AIC difference=7; Table 8). During the transition into middle school, the impact of mindfulness on self-perceived scholastic competence is attenuated compared to not transitioning. Therefore, someone with a higher mindfulness score will, on average, report a lower self-perceived scholastic competence score during the transition into middle school. See Figure 5 for depiction of the relationship between self-perceived scholastic competence, mindfulness and school transitions.

## Discussion

The present study investigated how self-perceived scholastic competence changes in adolescents over time, as well as how school transitions and mindfulness predict this aspect of self-perception over time. Our pre-registered multilevel analysis of 174 female adolescents, who contributed up to 3 waves of data, found no relationship between age and self-perceived scholastic competence. Instead, the data showed substantial variability in how self-perceived scholastic competence changed over time across participants, which aligns with previous findings identifying substantial individual variability in self-perceived scholastic competence during adolescence (e.g., Białecka-Pikul et al., 2019; Cole et al., 2001; Marsh & Martin, 2011). This contrasts with a study that assisted in forming our hypothesis that found a significant increase, on average, in self-perceived scholastic competence between 7th and 9th grade-aged individuals (Murdock et al., 2000). There are several differences between the present study and Murdock et al. (2000) including the age range examined, gender make-up of participants, the focus on school transition rather than age, and cohort (the data collected in the previous study are from 1993 and 1995). The variability in the present data suggests the importance of examining individual differences in self-perceived scholastic competence across adolescence.

We found evidence that, when controlling for prior levels of self-perceived scholastic competence, household income bracket, and age at menarche, the transitions into *both* middle school and high school predicted increases in self-perceived scholastic competence on average. This finding differs from our hypothesis that the transition into middle school would predict a decrease in self-perceived scholastic competence. Our observation of increasing self-perceived scholastic competence across school transitions is consistent with previous studies that suggest self-perceptions during childhood are low and increase during adolescence (Shavelson et al., 1976; Trzesniewski et al., 2003; Wiley, 1979). However, this finding is in contrast with findings from the studies that assisted in the development of the rationale for our hypothesis. Cantin and Boivin (2004) found that children experience a significant decrease in self-perceived scholastic competence after the transition from elementary to middle school, while Murdock et al. (2000) found that high schoolers report higher self-perceived scholastic competence than middle schoolers. There are several differences between these previous studies and the present study which could explain the discrepancy in the observed results, including different gender make-up of their sample and the inclusion of individuals in grades seven and nine. The present study examined both the transitions into middle school and high school (grades 4–11). We also included covariates such as socioeconomic status and age at menarche which could suggest these are confounding factors when examining the directionality of self-perceived scholastic competence at these transitions. When we included academic achievement in the model, the transition into middle school no longer significantly explained the variance we see in self-perception of scholastic competence in adolescence while the transition into high school remained a significant predictor. This could mean that academic achievement in the form of letter grades may contribute to the developmental trajectory of self-perceived scholastic competence more so during the transition from elementary to middle school. Overall, given evidence for changes in self-perception with school transitions rather than age, it may be that school transitions are an opportune window for intervention efforts to support positive self-perceptions.

Although a positive correlation between general self-perceptions and mindfulness has been previously observed (Bishop et al., 2004; Carlson, 2013), the relationship between *prior* levels of mindfulness and self-perceived *scholastic competence* in *adolescence* has not been previously explored. We hypothesized that higher prior levels of self-reported mindfulness would predict higher levels of self-perceived scholastic competence. This hypothesis was supported through multilevel modeling when controlling for prior levels of self-perceived scholastic competence, household income bracket, academic achievement, and age at menarche. Since there is a correlation between self-perceived scholastic competence and academic performance found in the present study, as well as previous studies (e.g., Guay et al., 2003; Marsh et al., 2005; Marsh & Martin, 2011), it is important to move forward with this research to increase rates of academic achievement among students. These findings show that incorporating mindfulness into these school-based interventions could be a promising approach for interventions aimed to improve self-perceptions of academic competence.

In a posthoc analysis, we saw that for every one point higher a participant scored on self-perceived scholastic competence, they were 23% more likely to receive an average letter grade of A as opposed to other grades (B, C, or not passing). The present study suggests that trait mindfulness may be a factor that can increase self-perceived scholastic competence. Knowing this, we can further explore the possible impact of mindfulness interventions on self-perceived scholastic competence to help support students in their academic success.

### Limitations & Future Directions.

The racial and ethnic diversity of the present sample was limited, specifically lacking in the representation of those from Asian, Black, and American Indian/Alaskan Native racial backgrounds. A more racially and ethnically diverse sample would improve the generalizability of these results. The data analyzed in the present report are from a longitudinal study that is still in progress, meaning not all the participants had completed all three waves of data. Future studies could explore possible gender differences in self-perceived scholastic competence, mindfulness, and school transitions. The present study used the Child and Adolescent Mindfulness Measure to assess the natural tendency one has to be mindful. In future studies, a measure assessing past and present experience with mindfulness practices could be beneficial to understanding the impact of engaging in mindfulness practices on self-perceived scholastic competence. Due to the expansion of the present analyses, we were unable to assess for a potential bidirectional effect of mindfulness and self-perceived scholastic competence. Therefore, we recommend future studies apply a structural equation modeling cross-lagged approach when examining this relationship. School transitions were assessed by change in grade across waves, meaning that not all participants were assessed immediately following their school transition. This leaves room for the student to possibly get settled, which can alter the stability of their self-perceptions (Wiley, 1979). Ideally, assessments of self-perceived scholastic competence would take place before each transition, shortly following the transition, and with long-term follow-up. In a posthoc analysis, academic achievement was assessed via self-reported average letter grade during an interview; this may not reflect accurate average academic achievement. In the future, accessing objective reports of grades (i.e., report cards), would help ensure accuracy. While the present study focused on person-level factors, another possible future direction of this work is to explore how school-level factors explain changes to self-perceived scholastic competence. This study did not consider school characteristics (private vs. public, general resources, size, etc.) due to limited access to information; however, they should be considered in the future.

## Conclusions

This study lays the groundwork to understand and support a crucial aspect of self-perception during adolescence—scholastic competence. This is important because self-perceived scholastic competence has been found to promote academic achievement (e.g., Guay et al., 2003; Marsh et al., 2005; Marsh & Martin, 2011; Sewasew & Schroeders, 2019) and occupational aspirations (Ireson & Hallam, 2009). While we did not find a relationship between age and self-perceived scholastic competence, we found evidence that trait-level mindfulness and school transitions predicted subsequently increased levels of self-perceived scholastic competence. We found that individuals with increased levels of self-perceived scholastic competence were more likely to have higher letter grades on average. We also found that individuals with higher levels of trait mindfulness, which can be increased further with mindfulness training (Carlson, 2013), reported increased levels of self-perceived scholastic competence. These two findings suggest a potential pathway from trait mindfulness to academic achievement through self-perceived scholastic competence. Another potential pathway could be that receiving better grades leads to an increase in self-perceived scholastic competence, and we recommend further work to help determine the directionality of these relationships.

This research can be expanded upon and should be, to identify and test malleable intervention targets at appropriate times within adolescence to improve self-perceived scholastic competence and to broadly support academic success during adolescence.

## Supplementary Material

Peer Review History

Supplemental Material

Peer Review History

Download: https://collabra.scholasticahq.com/article/57559-trait-mindfulness-supports-self-perceived-scholastic-competence-in-adolescent-girls/attachment/123126.docx?auth_token=zrTa6XgTOlIedgk32rr0

Supplemental Material

Download: https://collabra.scholasticahq.com/article/57559-trait-mindfulness-supports-self-perceived-scholastic-competence-in-adolescent-girls/attachment/123127.docx?auth_token=zrTa6XgTOlIedgk32rr0

## Figures and Tables

**Table 1.** Descriptive Statistics of Sample

	N	Percent of total
**Race and Ethnicity**		
Black or African American	4	2.31%
American Indian or Alaska Native	2	1.16%
Asian	2	1.16%
Native Hawaiian or Pacific Islander	0	0
Multi-Racial Hispanic/Latinx/Chicanx	4	2.31%
Non-Hispanic/Latinx/Chicanx White	119	68.79%
White Hispanic/Latinx/Chicanx	11	6.36%
Asian Hispanic/Latinx/Chicanx	0	0.00%
Black or African American Hispanic/Latinx/Chicanx	1	0.58%
Chicanx/Hispanic/Latinx Not Further Specified	8	4.62%
Non-Hispanic/Latinx/Chicanx Multi-Racial	22	12.72%
**Reported Family Income**		
Up to $25,000	19	10.92%
$25,000 to $40,000	26	14.94%
$40,000 to $75,000	40	22.99%
$75,000 to $100,000	34	19.54%
Over $100,000	40	22.99%
Don’t know	1	0.57%
Decline to respond	9	5.17%

**Table 2.** Missingness report of relevant measures

	Wave 1 (N=163)	Wave 2 (N=163)	Wave 3 (N=101)	Total (N=427)
**Transition**				
No Transition	82 (50.3%)	114 (69.9%)	38 (37.6%)	234 (54.8%)
Transitioned to Middle School	81 (49.7%)	43 (26.4%)	0 (0%)	124 (29.0%)
Transitioned to High School	0 (0%)	6 (3.7%)	63 (62.4%)	69 (16.2%)
**Age (years)**				
Mean (SD)	11.6 (0.810)	13.2 (0.821)	14.9 (0.602)	12.5 (1.33)
Median [Min, Max]	11.7 [9.98, 13.0]	13.3 [11.5, 14.9]	15.0 [13.7, 16.1]	12.3 [9.98, 16.1]
Missing	0 (0%)	16 (9.8%)	74 (73.3%)	90 (21.1%)
**Child and Adolescent Mindfulness Measure scores**				
Mean (SD)	26.0 (7.94)	25.5 (7.76)	23.2 (8.02)	25.2 (7.93)
Median [Min, Max]	27.0 [4.00, 40.0]	26.0 [0, 40.0]	22.0 [8.00, 40.0]	26.0 [0, 40.0]
Missing	42 (25.8%)	32 (19.6%)	36 (35.6%)	110 (25.8%)
**Self-Perceived Scholastic Competence**				
Mean (SD)	15.1 (3.48)	14.7 (3.68)	14.9 (3.24)	14.9 (3.51)
Median [Min, Max]	16.0 [5.00, 20.0]	15.0 [6.00, 20.0]	15.0 [6.00, 20.0]	15.0 [5.00, 20.0]
Missing	30 (18.4%)	28 (17.2%)	36 (35.6%)	94 (22.0%)

*Note*.

* Wave 3 contains a substantial amount of missing data due to the halt of data collection resulting from the COVID-19 pandemic

* If a participant is indicated as “Transitioned to Middle School” or “Transitioned to High School”, their transition took place during or before that wave of data collection and outside of the prior wave of data collection. See in text example above.

* This table reports on all participants who had their transition indicated. Participants were excluded from each wave if they did not have transition indicated.

* There are ages missing waves 2–3 because we do not have ages of participants who withdrew or skipped waves.

**Table 3.** Model comparisons and summary of self-perceived scholastic competence as predicted by school transitions

	*Dependent variable:*	
	Self-perceived Scholastic Competence	
	Without Transitions	With Transitions
	(1)	(2)
Low Socioeconomic Status	0.042 (0.691)	0.093 (0.671)
Middle Socioeconomic Status	−0.394 (0.758)	−0.596 (0.738)
Age at Menarche	0.010 (0.290)	0.028 (0.284)
Prior levels of Self-Perceived Scholastic Competence	0.606*** (0.088)	0.608*** (0.086)
Transition to High School		1.448** (0.638)
Transition to Middle School		2.662** (1.257)
Constant	5.616 (3.554)	4.825 (3.470)
Observations	106	106
Log Likelihood	−264.664	−260.348
Akaike Inf. Crit.	543.327	538.696

Note:

* p<0.1;

** p<0.05;

*** p<0.01

**Table 4.** Model comparisons and summary of self-perceived scholastic competence as predicted by prior levels of trait mindfulness

	*Dependent variable:*	
	Self-perceived Scholastic Competence	
	Without Mindfulness	With Mindfulness
	(1)	(2)
Low SES	0.067 (0.737)	−0.187 (0.730)
Middle SES	−0.598 (0.792)	−0.708 (0.780)
Age at Menarche	0.022 (0.305)	−0.107 (0.305)
Prior levels of SPPA-SC	0.577*** (0.091)	0.515*** (0.090)
Prior Levels of Trait Mindfulness		0.112*** (0.039)
Constant	6.064 (3.764)	5.846 (3.697)
Observations	100	100
Log Likelihood	−250.847	−246.746
Akaike Inf. Crit.	515.695	509.493

Note:

* p<0.1;

** p<0.05;

*** p<0.01

**Table 5.** Post-hoc analyses of self-perceived scholastic competence as predicted by contemporaneous levels of mindfulness

	*Dependent variable:*	
	Self-perceived Scholastic Competence	
	Without predictors	Only Contemporaneous
	(1)	(2)
Contemporaneous Levels of Mindfulness		0.118*** (0.034)
Constant	14.853*** (0.332)	11.965*** (0.896)
Observations	154	154
Log Likelihood	−405.354	−400.394
Akaike Inf. Crit.	816.709	808.788

Note:

* p<0.1;

** p<0.05;

*** p<0.01

**Table 6.** Post-hoc analyses of self-perceived scholastic competence predicted by prior levels of trait mindfulness while controlling for academic achievement

	*Dependent variable:*	
	Self-perceived Scholastic Competence	
	Without Mindfulness	With Mindfulness
	(1)	(2)
Low SES	0.518 (0.825)	0.176 (0.786)
Middle SES	−1.041 (0.835)	−1.237 (0.794)
Age at Menarche	0.263 (0.327)	0.108 (0.313)
Grades - B	−1.403* (0.725)	−1.599** (0.676)
Grades - C	−1.105 (1.474)	−1.555 (1.375)
Grades - Not Passing	−1.395 (3.051)	−1.378 (2.807)
Prior levels of SPPA-SC	0.490*** (0.097)	0.403*** (0.093)
Prior Levels of Trait Mindfulness		0.155*** (0.040)
Constant	4.878 (4.113)	4.393 (3.903)
Observations	94	94
Log Likelihood	−233.208	−225.548
Akaike Inf. Crit.	486.416	473.097

Note:

* p<0.1;

** p<0.05;

*** p<0.01

**Table 7.** Post-hoc analyses of self-perceived scholastic competence as predicted by school transitions while controlling for academic achievement

	*Dependent variable:*	
	Self-perceived Scholastic Competence	
	Without Transitions	With Transitions
	(1)	(2)
Low Socioeconomic Status	0.482 (0.764)	0.651 (0.764)
Middle Socioeconomic Status	−0.684 (0.771)	−0.872 (0.771)
Age at Menarche	0.185 (0.300)	0.166 (0.300)
Grades - B	−1.366* (0.695)	−1.478** (0.684)
Grades - C	−1.511 (1.329)	−1.641 (1.301)
Grades - Not Passing	−1.634 (2.168)	−1.447 (2.120)
Prior levels of Self-Perceived Scholastic Competence	0.540*** (0.092)	0.531*** (0.091)
Transition to High School		1.649** (0.657)
Transition to Middle School		1.547 (1.348)
Constant	4.909 (3.783)	4.786 (3.774)
Observations	100	100
Log Likelihood	−246.476	−242.875
Akaike Inf. Crit.	512.953	509.750

Note:

* p<0.1;

** p<0.05;

*** p<0.01

**Table 8.** Post-hoc analyses of self-perceived scholastic competence predicted by prior levels of trait mindfulness and school transitions

	*Dependent variable:*	
	Self-perceived Scholastic Competence	
	Without Interaction	With Interaction
	(1)	(2)
Low SES	−0.059 (0.724)	−0.223 (0.703)
Middle SES	−0.890 (0.771)	−1.011 (0.747)
Age at Menarche	−0.074 (0.304)	−0.041 (0.298)
Prior levels of SPPA-SC	0.513*** (0.089)	0.524*** (0.086)
High School Transition	1.319** (0.627)	3.897* (2.085)
Middle School Transition	2.643** (1.214)	12.919*** (3.495)
Prior Levels of Trait Mindfulness	0.105** (0.039)	0.167*** (0.046)
High School Transition * Prior Levels of Mindfulness		−0.101 (0.078)
Middle School Transition * Prior Levels of Mindfulness		−0.400*** (0.128)
Constant	5.111 (3.669)	3.060 (3.602)
Observations	100	100
Log Likelihood	−242.628	−237.217
Akaike Inf. Crit.	505.256	498.434

Note:

* p<0.1;

** p<0.05;

*** p<0.01

## Data Availability

Participant data and analysis scripts can be found on this paper’s project page on the provided link: https://osf.io/uynpm/

## Footnotes

1 1.7% of the sample at baseline did not identify as girls.

2 Recruitment targeted girls; at baseline, 1.7% of sample included participants who identified as non-binary.
