# Supplementary material for: Trait Mindfulness Supports Self-perceived Scholastic Competence in Adolescent Girls
Source: Collabra Psychol. Author manuscript; Available in PMC 2026 Apr 7. (PMC13052386; doi:10.1525/collabra.57559)
Supplement: Supplemental Material [file NIHMS2161622-supplement-Supplemental_Material.docx]

**Peer Review and Communication History**

**MS Title**: Trait Mindfulness Supports Self-perceived Scholastic Competence in Adolescent Girls

**Author Names**: Clare F. McCann, Theresa W. Cheng, Arian Mobasser, Jennifer H. Pfeifer, and Kathryn L. Mills

**Submitted:** Mar 1, 2022

**Editor First Decision**: Revise & Resubmit

Jun 2, 2022

Dear Clare F. McCann,

I have now received all reviews of your submission, “Trait mindfulness supports self-perceived scholastic competence in adolescent girls*”, from two very qualified researchers. I also independently read the manuscript. I agree that your manuscript has important strengths and also that there are some issues that need to be addressed. I therefore encourage you to submit a revised version for further consideration at Collabra: Psychology.

The reviewers did an outstanding job in their reviews. I will highlight issues I think are particularly salient here. In your resubmission, please include a document with a point-by-point response to both the points I list here and the reviewers’ comments, outlining each change made in your manuscript or providing a suitable rebuttal.

Please clarify the justification for the preregistered hypotheses and explain earlier how your study is connected to Greco (2011). Reviewer 1 also points out some important concerns with construct validity. Both reviews have some concerns with your statistical analyses that should be addressed. Finally, I agree with Reviewer 2 that you should examine whether the attrition is an issue, although attrition is understandable.

In summary, I think this is a promising manuscript and, I hope you will revise it for further consideration at Collabra: Psychology. I look forward to receiving your revision.

Please ensure that your revised files adhere to our author guidelines, and that the files are fully copyedited/proofed prior to upload. Please also ensure that all copyright permissions have been obtained. This is the last opportunity for major editing, therefore please fully check your file prior to resubmission.

If you have any questions or difficulties during this process, please contact the editorial office [editorialoffice@collabra.org](mailto:editorialoffice@collabra.org).

We hope you can submit your revision within the next six weeks. If you cannot make this deadline, please let us know as early as possible.

Sincerely,

Clare Conry-Murray

# Reviewer 1

##### Rating scale questions

|  | Strongly Disagree | Disagree | Neutral | Agree | Strongly Agree |
| --- | --- | --- | --- | --- | --- |
| The study/studies in this manuscript have strong construct validity (good measures and/or manipulations of the constructs the authors wish to study). (Choose “Neutral” if this is not an empirical manuscript) |  |  |  | ✔ |  |
| The study/studies in this manuscript have strong statistical validity (appropriate statistical tests, assumptions are clear and reasonable, no statistical errors, appropriate statistical inferences, etc.). (Choose “Neutral” if this is not an empirical manuscript) |  |  | ✔ |  |  |
| The study/studies in this manuscript have strong internal validity (any causal claims or implications are well-justified, alternative explanations are thoroughly considered, etc.). (Choose “Neutral” if this is not an empirical manuscript, or no causal claims are made or even vaguely implied.) |  | ✔ |  |  |  |
| The study/studies in this manuscript have strong external validity (authors appropriately constrain their conclusions based on the limits of the generalizability of their findings to other contexts (including from lab to real world), other populations, other stimuli or measures, etc.) |  |  |  | ✔ |  |

##### Open response questions

### Please write your review here. The author(s) will see this review. Your identity will not be revealed to the authors unless you also include your name (i.e., sign your review) in this box. It is up to you whether to reveal your identity or not, either is fine.

In the present work, the authors examine identity development in adolescents, and specifically consider – by means of a preregistered, longitudinal study across three waves – several questions relating to the shifting of individual differences across this time span. They found that scholastic competence increased from elementary to high school (i.e., that transitions would increase self-perceived competence), contrary to at least some past literature, and that mindfulness predicted better subsequent self-perceived self-perceived competence.
Overall, I think this is a good manuscript worthy of publication. I had several points I would like to see clarified, and some more macro concerns that I think could be addressed with a revised introduction, and perhaps dropping some analyses. I detail these below.

MAJOR

1. One aspect of the research that seems unclear from the introduction onwards is whether shifts in self-perceptions as children age/move through school systems are attributable to aging effects, or transition effects. Starting on page 3, I found that discussion of decreasing self-perceived academic competence from elementary to middle school, and increasing self-perceived competence from middle school to high school, was not clear whether these effects are happening because there is a self-competence low-point driven by the middle school age point, or if somehow these two transitions have opposite functions for children. The point about menarche (p. 4) seems to suggest either a biological side to this (or at least more than a pure “transition” effect), so I’m unclear how well past work tackles this distinction, and it should at least be mentioned (even if explained away) when talking about past work. It seems the prior work tackles this through its coding system of “school transition” (p. 11) which seems like a good compromise. But then I wasn’t sure how menarche became a covariate rather than a moderator (based on work showing menarche seems to moderate the influence of transitions, cited in intro).
2. (p. 6) I didn’t see how the preregistered hypothesis derived from the literature. It sounds like “self-perceived scholastic competence” decreases from elementary school to junior high, and then increases from junior high to high school. Indeed, the next paragraph says just this. Why does it follow “that self-perceived scholastic competence will increase on average from the time of elementary school to high school”? Seems like an unexplained assumption that the second transition will be bigger than the first. This may not be such a ‘major’ concern if just a clarity issue, but given it is a central hypothesis I found this fuzziness concerning.
3. The “final objective” is a prediction of mindfulness predicting higher levels of scholastic competence (I assume the authors mean self-perceived competence?), but then I don’t follow how this work goes beyond Greco et al. (2011) who they cite as discovering exactly this. If this work is intended as a preregistered (conceptual or otherwise) replication project that is great! But the introduction did not set me up to anticipate this was intended as a replication, so this could be clarified (or explained better, if the authors do not consider this project primarily a replication). This also speaks to contribution, though: why are these results interesting if Greco et al. already found them?
4. The statistics of this manuscript threw me off somewhat. For instance, on p. 15 it says that “Likelihood ratio tests comparing the full model to one excluding school transitions as a predictor show that including school transitions significantly improved the model without overfitting (χ2(3) =34.19, p<0.001, AIC difference=28; Table 3)”. But as one can tell from the 3 degrees of freedom, this is not a comparison of the full model versus one excluding school transitions. It appears to be a comparison between a full model and a model that drops two school transition variables and drops “prior levels of [self-perceived] scholastic competence.” Since the latter is (obviously) highly related to present scholastic competence, the comparison test seems biased. But I also wasn’t quite clear why this model comparison was going on, since all of the individual parameters are significant (see table 3) – so of course a model dropping three significant parameters would have to show worse fit than one that keeps all three highly significant predictors, right?
5. Also on results/consistency with the preregistration, the prereg stated that “Each model will use socioeconomic status (parent’s self-reported income) and pubertal onset (age at menarche) as covariates.” But then the manuscript proceeds to run analyses without those covariates: “When we examined the model without household income bracket and age-at-menarche, prior levels of mindfulness did not predict subsequent self-perceived scholastic competence”. I didn’t understand the point of this. You preregistered these covariates and it supported your results, so why run unplanned analyses without them that don’t work? This gets stranger (in my view) because on p. 21 this then forces you to run even more models to try to get back to significance by dropping even more variables. This ends up feeling like a lot of extra complexity / sends you down an analytical garden of forking paths, and makes the results untidy, despite the preregistered model supporting your key finding.
6. Overall, I found myself only really understanding your contribution by the discussion, but I think there should be more clarity about what you were trying to do to contribute by the end of the introduction.

MINOR

1. We are introduced to trait mindfulness on p. 4, and an emphasis is made on p. 5 that we are talking about trait rather than state. But in the same paragraph the authors focus us on “mindfulness is a potentially malleable factor” which seems more like they are interested in state mindfulness after all? Perhaps this could be clarified.
2. (p. 8) What does “prior to COVID-19” mean, precisely? I could imagine the pandemic having large effects on self-perceptions around academic competence/motivation, mindfulness, etc. If this collection was done months before the first warning signs of the virus, great – maybe just clarify that. However, it also states that “We included all available data up until data collection temporarily paused due to the impact of coronavirus for wave three” (p. 9) which sounds more like some data could have been influenced by the virus scare. The manuscript then states “from the participants who had completed sessions as of January 13th, 2020” which appears like a typo, but renders it unclear what this procedure was. Perhaps this was saying that the authors retained only data from sessions done before that date? In which case, a justification of why that specific date would be welcome.
3. I believe Harter’s (2012) scale has been well-validated, and that should be mentioned/cited. Ditto the other scales if they have been validated, too.
4. (p. 11) “Each participant’s CAMM score was calculated as the sum across items (range = 0–40). We used sum scores rather than mean scores to avoid including participants with mean scores that were derived from too few items.” I didn’t understand this logic at all. Why not just take the average but with listwise deletion of respondents who didn’t answer all items, or who didn’t answer some minimum of items? How does summing help?
5. (p. 13) “All betas reported are non-standardized”. So aren’t they Bs rather than betas? Betas are standardized by definition.
6. Should state, if true, that “no changes were made from the preregistration.” If not true, list all changes from the preregistration, please.
7. Why was only AIC used to select between models? If this is the only fit criterion available so be it, but perhaps state that. If there are other options (BIC is provided in the tables, why not used?), explain why they weren’t interpreted/used to inform decisions? Or if they agreed with AIC in all cases, say that: it provides more support for the inferences.
8. Figure 2 is very hard to make much of, even with the explanatory note. While I appreciate the authors wanting to be transparent/show all the data, this figure does not seem useful to me. Could it be replaced with something that makes the trends clearer? Figure 3 was much better.
9. (p. 19) Nice that these analyses were clearly stated as not part of the preregistration, that’s great. However, there also was not a lot of justification for why these analyses were worthwhile/interesting. That should be expanded on.
10. (p. 28) The authors frame this as though increases in mindfulness cause increases in scholastic competence which cause increases in academic performance. I.e., they recommend “the possible impact of mindfulness interventions on self-perceived scholastic competence to help support students in their academic success.” I was a bit unclear on how well the study’s chronology/design supports this conclusion, and this could be clarified in the discussion. What alternative pathways (e.g., getting good grades ◊ feeling competent; feeling competent ◊ mindfulness) can be ruled out based on design or theory?

# Reviewer 2

##### Rating scale questions

|  | Strongly Disagree | Disagree | Neutral | Agree | Strongly Agree |
| --- | --- | --- | --- | --- | --- |
| The study/studies in this manuscript have strong construct validity (good measures and/or manipulations of the constructs the authors wish to study). (Choose “Neutral” if this is not an empirical manuscript) |  |  | ✔ |  |  |
| The study/studies in this manuscript have strong statistical validity (appropriate statistical tests, assumptions are clear and reasonable, no statistical errors, appropriate statistical inferences, etc.). (Choose “Neutral” if this is not an empirical manuscript) |  |  | ✔ |  |  |
| The study/studies in this manuscript have strong internal validity (any causal claims or implications are well-justified, alternative explanations are thoroughly considered, etc.). (Choose “Neutral” if this is not an empirical manuscript, or no causal claims are made or even vaguely implied.) |  |  | ✔ |  |  |
| The study/studies in this manuscript have strong external validity (authors appropriately constrain their conclusions based on the limits of the generalizability of their findings to other contexts (including from lab to real world), other populations, other stimuli or measures, etc.) |  |  | ✔ |  |  |

##### Open response questions

### Please write your review here. The author(s) will see this review. Your identity will not be revealed to the authors unless you also include your name (i.e., sign your review) in this box. It is up to you whether to reveal your identity or not, either is fine.

The goal of the current study is to examine changes in self-perceived scholastic competence across a school transition and how changes related to mindfulness. The study includes data across three time points. The analyses were pre-registered and includes details about variations from the registration plan. Examining the link between mindfulness and self-perceived scholastic competence and school transitions is novel. However, more theoretical background and greater exploration of the data would strengthen the contribution.

1. It is not clear why mindfulness would be linked to changes in perceptions. What is the psychological process by which mindfulness relates to perceptions?
2. In addition, it is not clear why the study examines mindfulness and school transition. Is the hypothesis that mindfulness would buffer against declines in the transition to middle school and promote a greater increase in the transition to high school? The Introduction should explain why the three variables are included in the study and the processes by which they are linked.
3. The reason for the attrition due to COVID-19 is understandable and despite around 50% attrition can still be publishable. However, the extent of the bias in the attrition needs to be understood. Are there differences in the study variables for participants who remained in the study vs those who dropped out? How might these differences have impacted the findings of the study (e.g., those who would have declined in perceptions dropped out leading to the finding of no change).
4. Sum scores were used to avoid creating means from too few items. Does this mean that sums were calculated using different numbers of items (e.g., 3 items for one participant, 5 items for another)? This would mean that some participants have lower scores because they completed fewer items. Mean scores could be used, eliminating people who have fewer than x items per scale to address the stated concern. Or, given the high reliabilities of the scales, it is possible that including all participants would not introduce much bias in the data.
5. Please add a full correlation table.
6. It is interesting that when income and age of menarche are not included in the model, the role of mindfulness is reduced. Is there a large difference in the effect size or is it a situation of the effect size changing slightly to just below significance? If it is a large difference, what does this mean for understanding the role of mindfulness?
7. I wonder about the reverse effects of perceptions on mindfulness. Is there a bidirectional effect (e.g., cross-lag model) or might the relation found of mindfulness on perceptions actually reflect a reverse effect?
8. I know the sample size does not provide much power, but testing transition and mindfulness only as main effects feels like the test of two unrelated hypotheses. I was expecting an interaction between mindfulness and transition (see Point 2 above).
9. It is not clear to me what exactly is being tested in the models. Is mindfulness being regressed on both time points of perceptions?
10. I wonder whether it might be informative to stack the data, such that there are only two time points (e.g., baseline score can be Time 1 or Time 2 and follow-up is Time 2 or Time 3). This could add a little extra power and provide a straightforward analysis with the three groups (middle school, high school, no transition).
11. Overall, it is not clear to me what is being learned about self-perceptions.

**Author Response**
Aug 6, 2022

Hello,

My co-authors and I thank you for your email and the extremely helpful feedback for our manuscript “*Trait mindfulness supports self-perceived scholastic competence in adolescent girls**.” We appreciate the time granted to provide an updated manuscript addressing the following concerns and comments.

Below, we provide a point-by-point response to each comment with relevant revisions pasted for quick reference. The resubmitted manuscript includes tracked changes colored in red.

Please let us know if you need anything else!

**Reviewer 1**

Comment 1: One aspect of the research that seems unclear from the introduction onwards is whether shifts in self-perceptions as children age/move through school systems are attributable to aging effects, or transition effects. Starting on page 3, I found that discussion of decreasing self-perceived academic competence from elementary to middle school, and increasing self-perceived competence from middle school to high school, was not clear whether these effects are happening because there is a self-competence low-point driven by the middle school age point, or if somehow these two transitions have opposite functions for children. The point about menarche (p. 4) seems to suggest either a biological side to this (or at least more than a pure “transition” effect), so I’m unclear how well past work tackles this distinction, and it should at least be mentioned (even if explained away) when talking about past work. It seems the prior work tackles this through its coding system of “school transition” (p. 11) which seems like a good compromise. But then I wasn’t sure how menarche became a covariate rather than a moderator (based on work showing menarche seems to moderate the influence of transitions, cited in the intro).

Response 1: We thank Reviewer 1 for their thorough and thoughtful feedback on the present manuscript. We have made every effort in hopes of fulfilling the feedback received. We examined age effects in our first aim and found that age was not a significant predictor of self-perceived scholastic competence. We appreciate the need to clarify statements regarding trajectories of self-perceived scholastic competence in the introduction. Regarding menarche, originally, we were trying to keep this project streamlined and reduce the number of moderators investigated. However, we know the crucial role puberty is proven to play in identity formation and wanted to include it as a control.

**To address this we:**

- Clarified in the introduction on **page 3** our interpretation of the relevant literature. “In previous longitudinal studies, it has been found that children and younger adolescents experience a significant decrease in self-perceived academic competence after the transition from elementary to middle school over and above age at the time of self-report (typically after grades 5-7; e.g., Cantin and Boivin 2004; Coelho and Romão 2017; Cole et al. 2001; similar findings have come from cross-sectional studies such as Onetti et al. 2019).”
- Added a post hoc analysis looking at menarche as a moderator. We found that adding age at menarche as a moderator did not improve model fit and the effect was non-significant. **(Page 24)** “Previous literature suggests puberty may play a role in female adolescent’s self-perception trajectories during the transition from elementary to middle and middle to high school. To account for this, we ran a post-hoc analysis examining age at menarche as a moderator of the relationship between school transitions and self-perceived scholastic competence. We found that the addition of the age at menarche to the full model, including SES, school transitions, and prior levels of self-perceived scholastic competence did not improve the model fit, and the effect was not significant.”

| **Self-perceived scholastic competence predicted by school transitions moderated by age at menarche** | | | |
| --- | --- | --- | --- |
|  | | | |
|  | *Dependent variable:* | | |
|  |  | | |
|  | Self-perceived Scholastic Competence | | |
|  | Without Predictors | Without Age at Menarche | With Age at Menarche |
|  | (1) | (2) | (3) |
|  | | | |
| Low SES | -0.509 (0.996) | 0.091 (0.668) | 0.093 (0.671) |
| Middle SES | -1.510 (1.038) | -0.577 (0.710) | -0.596 (0.738) |
| Age at Menarche |  |  | 0.028 (0.284) |
| Prior levels of SPPA-SC |  | 0.610^***^ (0.083) | 0.608^***^ (0.086) |
| High School Transition |  | 1.453^**^ (0.634) | 1.448^**^ (0.638) |
| Middle School Transition |  | 2.648^**^ (1.243) | 2.662^**^ (1.257) |
| Constant | 15.078^***^ (0.629) | 5.135^***^ (1.349) | 4.825 (3.470) |
|  | | | |
| Observations | 106 | 106 | 106 |
| Log Likelihood | -278.493 | -260.353 | -260.348 |
| Akaike Inf. Crit. | 566.986 | 536.706 | 538.696 |
| Bayesian Inf. Crit. | 580.303 | 558.014 | 562.667 |
|  | | | |
| *Note:* | ^*^p^<0.1; >**^p^<0.05; >***^p<0.01 | | |

Comment 2: (p. 6) I didn’t see *how the preregistered hypothesis was derived from the literature*. It sounds like “self-perceived scholastic competence” decreases from elementary school to junior high, and then increases from junior high to high school. Indeed, the next paragraph says just this. Why does it follow “that self-perceived scholastic competence will increase on average from the time of elementary school to high school”? Seems like an unexplained assumption that the second transition will be bigger than the first. This may not be such a ‘major’ concern if just a clarity issue, but given it is a central hypothesis I found this fuzziness concerning.

Response 2: Because we examined age effects first, we hypothesized that over the span of elementary age to high school age, there would be a general increase as shown in previous work.

To address this we added the following to **page 6**: “The first objective of this study is to examine how self-perceived scholastic competence changes across time in adolescent girls. Due to prior literature reviewed above, we hypothesize that self-perceived scholastic competence will increase on average suggesting that the increase from middle school to high school surpasses the original decrease following elementary school.”

Comment 3: The “final objective” is a prediction of mindfulness predicting higher levels of scholastic competence (I assume the authors mean self-perceived competence?), but then I don’t follow how this work goes beyond Greco et al. (2011) who they cite as discovering exactly this. If this work is intended as a preregistered (conceptual or otherwise) replication project that is great! But the introduction did not set me up to anticipate this was intended as a replication, so this could be clarified (or explained better, if the authors do not consider this project primarily a replication). This also speaks to the contribution, though: why are these results interesting if Greco et al. already found them?

Response 3: Greco et al. focused on academic competence in the context of school reports. The present study focuses on self-perceived scholastic competence (how one feels regarding their performance in school, not their actual performance). Therefore, the two studies are focusing on two related, but distinct constructs. We draw from the findings reported in Greco et al. to make the argument for investigating mindfulness and academic functioning, and suggest self-perceived scholastic competence could serve as a potential mechanism for this relationship. We’ve now clarified on **page 7** that Greco et al specifically identified higher academic functioning in the form of school reports.

Comment 4: The statistics of this manuscript threw me off somewhat. For instance, on p. 15 it says that “Likelihood ratio tests comparing the full model to one excluding school transitions as a predictor show that including school transitions significantly improved the model without overfitting (χ2(3) =34.19, p<0.001, AIC difference=28; Table 3)”. But as one can tell from the 3 degrees of freedom, this is not a comparison of the full model versus one excluding school transitions. It appears to be a comparison between a full model and a model that drops two school transition variables and drops “prior levels of [self-perceived] scholastic competence.” Since the latter is (obviously) highly related to present scholastic competence, the comparison test seems biased. But I also wasn’t quite clear why this model comparison was going on, since all of the individual parameters are significant (see table 3) – so of course a model dropping three significant parameters would have to show worse fit than one that keeps all three highly significant predictors, right?

Response 4: We wanted to show that school transitions were significant predictors of self-perceived scholastic competence above and beyond our covariates. The reason the degrees of freedom is equal to 3 is because of the addition of prior levels of self-perceived scholastic competence as well. To avoid confusion, we have altered the sentence to avoid redundancy in our description of the model comparison procedure.

- **To address this**, we amended the following on **page 18**: “Likelihood ratio tests comparing the full model to one excluding prior mindfulness scores as a predictor shows that including prior mindfulness scores significantly improved the model without overfitting.”

Comment 5: Also on results/consistency with the preregistration, the preregistration stated that “Each model will use socioeconomic status (parent’s self-reported income) and pubertal onset (age at menarche) as covariates.” But then the manuscript proceeds to run analyses without those covariates: “When we examined the model without household income bracket and age-at-menarche, prior levels of mindfulness did not predict subsequent self-perceived scholastic competence”. I didn’t understand the point of this. *You preregistered these covariates and it supported your results, so why run unplanned analyses without them that don’t work?* This gets stranger (in my view) because on p. 21 this then forces you to run even more models to try to get back to significance by dropping even more variables. This ends up feeling like a lot of extra complexity / sends you down an analytical garden of forking paths, and makes the results untidy, despite the preregistered model supporting your key finding.

Response 5: Despite pre-registering the use of socioeconomic status and pubertal onset as covariates, we initially reported analyses that did not include these covariates due to a higher proportion of missing data on these variables that greatly reduced our sample size. However, we agree with the reviewer that it is important to focus on reporting pre-registered analyses.

- **To address this**: We have moved models excluding the covariates into the supplementary information on the larger sample.

Comment 6: Overall, I found myself only really understanding your contribution by the discussion, but I think there should be more clarity about what you were trying to do to contribute by the end of the introduction.

Response 6: We have also included sentences from the introduction below that we feel contribute to the overarching comment regarding the contributions in the introduction.

- **(Page 2)** Since academic self-concept is tied to academic achievement, it is important to identify the factors relating to the development of this facet of identity.
- **(Page 2)** By characterizing developmental changes in self-perceived scholastic competence in adolescence, we can better understand how this aspect of self-concept changes on average, as well as how variable this change is across individuals.
- **(Page 2-3)** The current study investigates these two factors in a longitudinal study to specify how transitions into middle school, and levels of trait mindfulness, relate to the self-perception of scholastic competence in adolescent girls, who have been found to be at increased risk for more negative self-concepts.
- **(Page 3)** The work summarized here tells us that we know shifts in self-perception of scholastic competence occur with school transitions, but the directionality of this instability needs further examination.
- **(Page 4)** Therefore, further addressing the impact of both middle school and high school transitions in a longitudinal sample consisting of female adolescents, would help expand our understanding of how academic self-perceptions change over time for girls. Further, distinguishing the impact of the transition into middle school as compared to the transition into high school on self-perceived scholastic competence is needed to understand the unique challenges of transitions in hopes to promote more positive self-perceived scholastic competence during the early-to-mid adolescent period.
- **(Page 5-6)** To help lay a foundation for understanding how mindfulness and self-perceived scholastic competence relate across adolescence, we need to assess whether trait mindfulness is a malleable factor promoting positive academic self-perception in a longitudinal study. By doing so, we can possibly increase the motivation to implement mindfulness learning.

Comment 7: We are introduced to trait mindfulness on p. 4, and an emphasis is made on p. 5 that we are talking about trait rather than state. But in the same paragraph the authors focus us on “mindfulness is a potentially malleable factor” which seems more like they are interested in state mindfulness after all? Perhaps this could be clarified.

Response 7: You are correct in that we examine trait mindfulness. Trait mindfulness can be increased through mindfulness training. Therefore, trait mindfulness is malleable. Please see below to see how we have clarified in the manuscript.

- **To address this** we added the following to the intro on **page 5**: “In the present study, we examine trait mindfulness, which can be increased with training (Carlson, 2013), and its relationship to self-perceived scholastic competence. Among adolescents, increasing levels of mindfulness are related to higher academic functioning (Greco et al. 2011) and stronger global self-worth (Brown and Ryan 2003). Expressing and implementing mindfulness in the school-based context is recognized by educators as being associated with fostering attention, resilience, and well-being (*Garrison Institute, 2005*)*.* These findings suggest that mindfulness is a potentially malleable factor related to academic achievement, self-perceptions, and well-being. Due to the malleable nature of trait mindfulness, we propose gaining further understanding of the relationship between trait mindfulness and self-perceived scholastic competence could have implications in improving how individuals feel about themselves in an academic setting. For example, if higher levels of trait mindfulness is found to be related to increased self-perceived scholastic competence than future work could focus on improving trait mindfulness to mimic this effect.”

Comment 8: (p. 8) What does “prior to COVID-19” mean, precisely? I could imagine the pandemic having large effects on self-perceptions around academic competence/motivation, mindfulness, etc. If this collection was done months before the first warning signs of the virus, great – maybe just clarify that. However, it also states that “We included all available data up until data collection temporarily paused due to the impact of coronavirus for wave three” (p. 9) which sounds more like some data could have been influenced by the virus scare. The manuscript then states “from the participants who had completed sessions as of January 13th, 2020” which appears like a typo, but renders it unclear what this procedure was. Perhaps this was saying that the authors retained only data from sessions done before that date? In which case, a justification of why that specific date would be welcome.

Response 8: In the context of this longitudinal study, prior to COVID-19 means any data collected before January 13th, 2020. Therefore, the data collected would have not been impacted by the effects of the COVID-19 scare or lockdown.

- **To address this** we have added the following to **page 9**: “The sample size at the third wave (N=101) is not true attrition due to individuals dropping out, it was based on the schedule of initial recruitment. We used data collected up until January 13th, 2020 (pre-COVID scare and lockdown) for simplicity and to meet necessary timelines for the first author’s degree requirements. Data collection was paused in our lab for 8-9 months, after which it resumed and approximately 60 more participants completed a third wave of data collection. The degree of attrition over each wave in the study has averaged ~ 5%. Regarding bias in the sample, we have observed that the age at initial recruitment tended to decrease over the duration of the first wave (participants in older grade levels were recruited first until we expanded to include a younger grade level, even though the age range for initial inclusion did not change). Therefore, it is likely that some of the very youngest participants in the study overall provided data for the third wave after the pandemic, and those are the participants whose data are missing at the third wave. Finally, using data collected after the pandemic, given the extensive disruptions to schooling and in-person education, is interesting but complex for those reasons; therefore, it best represents a promising future direction for additional research.”

Comment 9: I believe Harter’s (2012) scale has been well-validated, and that should be mentioned/cited. Ditto the other scales if they have been validated, too.

Response 9: Thank you for pointing this out! We will definitely include this.

- **To address this**, we added: “well-validated” before each measure name and cited the relevant literature.

Comment 10: (p. 11) “Each participant’s CAMM score was calculated as the sum across items (range = 0–40). We used sum scores rather than mean scores to avoid including participants with mean scores that were derived from too few items.” I didn’t understand this logic at all. Why not just take the average but with listwise deletion of respondents who didn’t answer all items, or who didn’t answer some minimum of items? How does summing help?

Response 10: We apologize for the confusion. We agree that summing might not necessarily help, but we were using the sum score as this procedure is consistent with the manual for this measure. We have **now clarified in the text** that we are summing in accordance with Greco et al, 2011.

Comment 11: (p. 13) “All betas reported are non-standardized”. So aren’t they Bs rather than betas? Betas are standardized by definition.

Response 11: We apologize for the confusion. We have **now updated our language** and symbols to clarify that we are using unstandardized coefficients.

Comment 12: Should state, if true, that “no changes were made from the preregistration.” If not true, list all changes from the preregistration, please.

Response 12: All non-preregistered analyses fall under “**Deviations from Pre-Registration**” starting on **page 19** or are included in the supplementary material.

Comment 13: Why was only AIC used to select between models? If this is the only fit criterion available so be it, but perhaps state that. If there are other options (BIC is provided in the tables, why not used?), explain why they weren’t interpreted/used to inform decisions? Or if they agreed with AIC in all cases, say that: it provides more support for the inferences.

Response 13: We state that our usage of AIC in reporting model comparison statistics is standard lab operating procedure but have removed BIC from our tables to avoid reporting confusion. We use AIC because it’s been shown to be sufficient in assessing for overfitting.

- **To address this,** we removed BIC from our tables.
- Here is a list of citations using AIC from our lab to demonstrate the standard operating procedure:

i. Vijayakumar et al., 2020; Journal of Personality Social Psychology.
ii. Andrews et al., 2020; Journal of Research on Adolescence.
iii. Vijayakumar et al., 2020; Cortex.
iv. Mills et al., 2016; NeuroImage.

Comment 14: Figure 2 is very hard to make much of, even with the explanatory note. While I appreciate the authors wanting to be transparent/show all the data, this figure does not seem useful to me. Could it be replaced with something that makes the trends clearer? Figure 3 was much better.

Response 14: Thank you for letting us know. However, we feel that displaying the effects of school transition in this way best allows us to account for the relationship between the covariates and the outcome. We have **updated the figure caption** to facilitate ease of interpretation better.

Comment 15: (p. 19) Nice that these analyses were clearly stated as not part of the preregistration, that’s great. However, there also was not a lot of justification for why these analyses were worthwhile/interesting. That should be expanded on.

Response 15: We state on **page 19**, “The nature of these post-hoc analyses was (a) to interrogate how including or not including time-lagged and contemporaneous versions of dependent (self-perceived scholastic competence) and independent (levels of mindfulness) variables impacted our findings, and (b) to investigate more objective measures of scholastic competence (academic achievement).”

Comment 16: (p. 28) The authors frame this as though increases in mindfulness cause increases in scholastic competence which causes increases in academic performance. I.e., they recommend “the possible impact of mindfulness interventions on self-perceived scholastic competence to help support students in their academic success.” I was a bit unclear on how well the study’s chronology/design supports this conclusion, and this could be clarified in the discussion. What alternative pathways (e.g., getting good grades feeling competent; feeling competent mindfulness) can be ruled out based on design or theory?

Response 16: This is a fair point. We have done the following in hopes of softening our conclusion.

- **To address this** we add the following to **page 30**: “We found that individuals with increased levels of self-perceived scholastic competence were more likely to have higher letter grades on average. We also found that individuals with higher levels of trait mindfulness, which can be increased further with mindfulness training (Carlson, 2013), reported increased levels of self-perceived scholastic competence. These findings suggest a potential pathway from trait mindfulness to academic achievement through self-perceived scholastic competence. Another potential pathway could be that receiving better grades leads to increased competence, and we recommend further work to help determine the directionality of these relationships.”

**Reviewer 2**

Comment 17: It is not clear why mindfulness would be linked to changes in perceptions. What is the psychological process by which mindfulness relates to perceptions?

Response 17: We have **added the following** to the intro on **page 5**: “mindfulness reduces reactivity and defensiveness toward ego-threatening information, which should reduce the tendency to process information about one’s personality in biased ways.”

Comment 18: In addition, it is not clear why the study examines mindfulness and school transition. Is the hypothesis that mindfulness would buffer against declines in the transition to middle school and promote a greater increase in the transition to high school? The Introduction should explain why the three variables are included in the study and the processes by which they are linked.

Response 18: Thank you for bringing this up. The original intent of including trait mindfulness and school transitions was to uncover potential mechanisms driving the instability in self-perceptions across development. As stated in the introduction, prior research suggests both mindfulness and school transitions impactcting the way one perceives themselves. We believe that the addition of the interaction model, as suggested in comment 24, helps tie the two processes together.

- To address this, we have added the following to **page 6**: “The present study examines both trait mindfulness and school transitions as both separate potential mechanisms contributing to the instability in self-perceptions across adolescence, and later on examine them together.”
- Add the interaction results and explanation to **page 26**.

Comment 19: The reason for the attrition due to COVID-19 is understandable and despite around 50% attrition can still be publishable. However, the extent of the bias in the attrition needs to be understood. Are there differences in the study variables for participants who remained in the study vs those who dropped out? How might these differences have impacted the findings of the study (e.g., those who would have declined in perceptions dropped out leading to the finding of no change).

Response 19: Thank you for bringing this up.

- To address this, we have added the following to **page 9** in the manuscript: The sample size at the third wave is not true attrition due to individuals dropping out, it was based on the schedule of initial recruitment. We used data collected up until January 13th, 2020 (pre-COVID scare and lockdown) for simplicity and to meet necessary timelines for the first author’s degree requirements. Data collection was paused in our lab for 8-9 months, after which it resumed and approximately 60 more participants completed a third wave of data collection. The degree of attrition over each wave in the study has averaged ~ 5%. Regarding bias in the sample, we have observed that the age at initial recruitment tended to decrease over the duration of the first wave (participants in older grade levels were recruited first until we expanded to include a younger grade level, even though the age range for initial inclusion did not change). Therefore it is likely that some of the very youngest participants in the study overall provided data for the third wave *after* the pandemic, and those are the participants whose data are missing at the third wave. Finally, using data collected after the pandemic, given the extensive disruptions to schooling and in-person education, is interesting but complex for those reasons; therefore, it best represents a promising future direction for additional research.

Comment 20: Sum scores were used to avoid creating means from too few items. Does this mean that sums were calculated using different numbers of items (e.g., 3 items for one participant, 5 items for another)? This would mean that some participants have lower scores because they completed fewer items. Mean scores could be used, eliminating people who have fewer than x items per scale to address the stated concern. Or, given the high reliabilities of the scales, it is possible that including all participants would not introduce much bias in the data.

Response 20: Please see our above reply to **comment 4**.

Comment 21: Please add a full correlation table.

Response 21: Thank you for this suggestion. We have now included a full correlation table in the supplementary material. You can view it below:

Comment 22: It is interesting that when income and age of menarche are not included in the model, the role of mindfulness is reduced. Is there a large difference in the effect size or is it a situation of the effect size changing slightly to just below significance? If it is a large difference, what does this mean for understanding the role of mindfulness?

Response 22: This is a fair question. When examined without covariates, we found that mindfulness had an unstandardized effect size of 0.04 with a p-value of 0.175. With the covariates, mindfulness had an unstandardized effect size of 0.11 with a p-value of 0.008. This 0.07 change in effect size could suggest that the addition of the covariates do matter since the effect size of mindfulness becomes stronger when looking at the model with the covariates. We removed all analyses without covariates to avoid confusion per comment 5.

- **To address this** we added the above figure to **supplementary material**.

Comment 23: I wonder about the reverse effects of perceptions on mindfulness. Is there a bidirectional effect (e.g., cross-lag model), or might the relation found of mindfulness on actually reflect a reverse effect?

Response 23: The current pre-registered study aimed to examine one direction only, and although intriguing we do not know of any literature that would suggest a reverse effect. Due to the large number of additional analyses that have already been added to the manuscript, we addressed this issue by commenting on the possibility of examining the reverse path, as well as cross-lagged paths in SEM, in the section on future directions.

Comment 24: I know the sample size does not provide much power, but testing transition and mindfulness only as main effects feels like the test of two unrelated hypotheses. I was expecting an interaction between mindfulness and transition (see Point 2 above).

Response 24: We have added a posthoc analysis to **page 25** using an interaction between mindfulness and transitions.

“Finally, we wanted to explore the potential relationship between school transitions, mindfulness, and self-perceived scholastic competence all together. As both mindfulness and school transitions are significant predictors of self-perceived scholastic competence, we examined their interaction as a potential moderator of these relationships. We found that the inclusion of the interaction between prior levels of mindfulness and school transitions significantly improved model fit when added to a model including our covariates, prior levels of mindfulness, school transitions and a random intercept (χ2(12) =10.82, p=0.005, AIC difference=7). During the transition into middle school, the impact of mindfulness on self-perceived scholastic competence is attenuated compared to not transitioning. Therefore, trait mindfulness’ impact may be higher when someone is not transitioning.”

| **Self-perceived scholastic competence predicted by prior levels of trait mindfulness and school transitions** | | |
| --- | --- | --- |
|  | | |
|  | *Dependent variable:* | |
|  |  | |
|  | Self-perceived Scholastic Competence | |
|  | Without Interaction | With Interaction |
|  | (1) | (2) |
|  | | |
| Low SES | -0.059 (0.724) | -0.223 (0.703) |
| Middle SES | -0.890 (0.771) | -1.011 (0.747) |
| Age at Menarche | -0.074 (0.304) | -0.041 (0.298) |
| Prior levels of SPPA-SC | 0.513^***^ (0.089) | 0.524^***^ (0.086) |
| High School Transition | 1.319^**^ (0.627) | 3.897^*^ (2.085) |
| Middle School Transition | 2.643^**^ (1.214) | 12.919^***^ (3.495) |
| Prior Levels of Trait Mindfulness | 0.105^**^ (0.039) | 0.167^***^ (0.046) |
| High School Transition * Prior Levels of Mindfulness |  | -0.101 (0.078) |
| Middle School Transition * Prior Levels of Mindfulness |  | -0.400^***^ (0.128) |
| Constant | 5.111 (3.669) | 3.060 (3.602) |
|  | | |
| Observations | 100 | 100 |
| Log Likelihood | -242.628 | -237.217 |
| Akaike Inf. Crit. | 505.256 | 498.434 |
|  | | |
| *Note:* | ^*^p^<0.1; >**^p^<0.05; >***^p<0.01 | |

**Fig 5.** Plot of Simple Slopes from a Model Including an Interaction Between School Transitions and Mindfulness. The y-axis is the self-perceived scholastic competence score, and the x-axis is the prior level of mindfulness. The blue continuous line represents the simple slope for those that do not transition, the pink long dashed line represents the simple slope for those that transitioned into high school, and the green dotted line represents the simple slope for those that transitioned into middle school.

Comment 25: It is not clear to me what exactly is being tested in the models. Is mindfulness being regressed on both time points of perceptions?

Response 25: Yes, we used **multilevel modeling** to examine the relationship. Please view the equation below.

We have added the following to **page 14** of the manuscript:

“Here is an example of a multilevel model: *SPPA-SC_ij_ = B_0j_ + B_1j_Mindfulness_ij_ + e*_ij_. *SPPA-SC_ij_* represents the dependent variable which is self-perceived scholastic competence for the present study. The “*i*” subscript represents the score at a certain level or time point, and the “*j*” subscript represents the group which is each participant for the present study. Mindfulness_ij_ represents the level 1 predictor, *B_0j_* is the intercept of the dependent variable in group “*j*” or level 2, *B_1j_* refers to the slope for the relationship in group “*j*” or level 2 between the level 1 predictors and the dependent variable. *e_ij_* represents the random errors of prediction for the level 1 equation.” You can also reference our publicly available code here: <https://github.com/clarefmccann/project-1>

Comment 26: I wonder whether it might be informative to stack the data, such that there are only two time points (e.g., baseline score can be Time 1 or Time 2 and follow-up is Time 2 or Time 3). This could add a little extra power and provide a straightforward analysis with the three groups (middle school, high school, no transition).

Response 26: Our current approach effectively uses the principles described by the reviewer above; in the pre-registered analysis, each participant effectively contributed up to two separate rows of data due to the lagged independent variable of interest.

**Editor First Decision**: Revise & Resubmit

Sep 20, 2022

Dear Dr. McCann,

I have now received one review of your manuscript, “Trait mindfulness supports self-perceived scholastic competence in adolescent girls*” and I also independently read the manuscript. The reviewer had mostly positive reactions to your manuscript and I agree that your manuscript has important strengths and also that there are some issues that need to be addressed. I therefore encourage you to submit a revised version for further consideration at Collabra: Psychology.

The reviewer did an outstanding job in their reviews. I will highlight issues I think are particularly salient here. In your resubmission, please include a document with a point-by-point response to both the points I list here and the reviewers’ comments, outlining each change made in your manuscript or providing a suitable rebuttal.

Major revisions:
Address the issue with model comparisons bright up by the reviewer, as well as the other concerns.

Minor revisions:
Add citations for the idea that trat mindfulness is malleable (stated several tims on p. 5), rather than just different in different individuals. Was Carlson, 2013 not supposed to be crossed out? If past research does not show this, then make it clearer that this needs to be tested even though it might logically follow from current understandings.

Why the focus on girls? This needs to be addressed more explicitly, though it may not be a big change. Page 4 discusses gender a but, but a sentence stating, “We focus on girls because…” would be helpful.

Figure 2 is still unclear.

In summary, I think this is a promising manuscript and, I hope you will revise it for further consideration at Collabra: Psychology. I look forward to receiving your revision. Please see the instructions below for submitting your revision.

Please ensure that your revised files adhere to our author guidelines, and that the files are fully copyedited/proofed prior to upload. Please also ensure that all copyright permissions have been obtained. This may be the last opportunity for major editing, therefore please fully check your file prior to re-submission.

If you have any questions or difficulties during this process, please contact the editorial office at [editorialoffice@collabra.org](mailto:editorialoffice@collabra.org).

We hope you can submit your revision within the next six weeks. If you cannot make this deadline, please let us know as early as possible.

Sincerely,

Clare Conry-Murray

# Reviewer 1

##### Rating scale questions

|  | Strongly Disagree | Disagree | Neutral | Agree | Strongly Agree |
| --- | --- | --- | --- | --- | --- |
| The study/studies in this manuscript have strong construct validity (good measures and/or manipulations of the constructs the authors wish to study). (Choose “Neutral” if this is not an empirical manuscript) |  |  |  |  | ✔ |
| The study/studies in this manuscript have strong statistical validity (appropriate statistical tests, assumptions are clear and reasonable, no statistical errors, appropriate statistical inferences, etc.). (Choose “Neutral” if this is not an empirical manuscript) |  |  | ✔ |  |  |
| The study/studies in this manuscript have strong internal validity (any causal claims or implications are well-justified, alternative explanations are thoroughly considered, etc.). (Choose “Neutral” if this is not an empirical manuscript, or no causal claims are made or even vaguely implied.) |  | ✔ |  |  |  |
| The study/studies in this manuscript have strong external validity (authors appropriately constrain their conclusions based on the limits of the generalizability of their findings to other contexts (including from lab to real world), other populations, other stimuli or measures, etc.) |  |  |  | ✔ |  |

##### Open response questions

### Please write your review here. The author(s) will see this review. Your identity will not be revealed to the authors unless you also include your name (i.e., sign your review) in this box. It is up to you whether to reveal your identity or not, either is fine.

I thank the authors for their careful attention to the comments submitted by myself and the other peer reviewer. Overall, I am personally much happier with the revised version of the manuscript. If I do not reference a comment, it can be assumed I was okay with the authors’ response. Beyond that, in most cases I thought the changes were really good and tackled the desired changes nicely.

Comment 4—
I still think that the comparisons between ‘with transitions’ and ‘without transitions’ models (various Tables) should include “Prior levels of Self-Perceived Scholastic Competence” in the ‘without XXX’ (e.g., ‘without transitions’ in Table 3) model, too. Otherwise, the comparison between models artificially is inflated by its addition in only the ‘with transitions’ model. Let me put it this way: imagine that transitions did nothing. The ‘with transitions’ model might show better fit (AIC) purely because it adds the pretest of SPPC in the model, right? So I would just put the pretest of SPPC in BOTH models, so that the comparison of models statistics can refer exclusively to the ‘upgrade’ due to transitions.

Same comment for Table 4, but substituting ‘mindfulness’ for ‘transitions’. Once again, SPPC pretest should also be in the ‘without mindfulness’ model/column.

Same comment for Tables 6-7, and any other table where pretest of SPPC is excluded from the reduced (left) models.

Comment 10 — Two issues here. First, the phrasing about using sums because of the perceived issue with means was not just used for the mindfulness measure, but also for the SPPC measure, but no revision was made for the latter.

Second, I would simply recommend DROPPING the language about using sums to avoid means from participants with too little data. I get that there’s another justification (which makes sense: it’s what the scale authors want). But THIS explanation is a red herring and is therefore confusing. I also note Reviewer 2 was confused by this.

I think the authors mean the following: “using our software’s SUM function means that participants with
missing data on a given scale are listwise deleted for that scale, which we want. We didn’t want to use a MEAN-based function because it would calculate means even for respondents with some missing data, which we see
as problematic.” But the thing is, this explanation makes no sense because you can just as easily calculate mean scores that drop participants who didn’t answer X% of items (see the sjstats package for R). So I would really just recommend dropping this extra bit, as it’s confusing.

Comment 14— Ultimately, this is the editor’s call. But I’m still finding this figure #2 really puzzling. I am not a developmental psychologist and so can appreciate if I’m being dense. Perhaps this is a more common type of figure in longitudinal analyses. However, I simply do not understand what it shows. The revised caption only partially helps.

“The purple box represents the transition into
middle school, the orange box represents no transition, and the blue box represents the
transition into high school. The line in the middle of each box is the effect of each transition
or non-transition on self-perceived scholastic competence.”

What do the bars indicate, /exactly/? Saying they “represent the transition” or that they are “the effect” is not helping me understand. Are those means?

I’d once again just dropping the figure, as I don’t see what it adds beyond the tables. This isn’t an interaction - it’s simply first-order “main” effects (I think). Does it even need a figure? Could the main text unpack it, if it very important to communicate something?

Again, apologies that I’m not ‘getting it’ and I am not trying to be difficult. But I do suspect that some readers would share my confusion.

Comment 16—

I am good with the response, but just noted this minor detail. Here is the quote:

“Another potential pathway could be that receiving
better grades leads to increased competence, and we recommend further work to help
determine the directionality of these relationships”

I’ve mentioned before, but really all references to ‘competence’ that mean ‘self-perceived competence’ should say the latter. This is particularly problematic because the authors do use actual grades at some points, which could be mistaken as being ‘competence’. Self-perceived competence makes this unambiguous.

**Author Response**
Oct 14, 2022

Hello,

My co-authors and I thank you for your email and the extremely helpful feedback for our manuscript “*Trait mindfulness supports self-perceived scholastic competence in adolescent girls**.” We appreciate the time granted to provide an updated manuscript addressing the following concerns and comments.

Below, we provide a point-by-point response to each comment with relevant revisions pasted for quick reference. The resubmitted manuscript includes tracked changes colored in red.

Please let us know if you need anything else!

**Editor**

- Address the issue with model comparisons brought up by the reviewer, as well as the other concerns.
  - See response to reviewer 1’s Comment 1.
- Figure 2 is still unclear.
  - See response to reviewer 1’s Comment 4.

**Reviewer 1**

**Comment 1:** I still think that the comparisons between ‘with transitions’ and ‘without transitions’ models (various Tables) should include “Prior levels of Self-Perceived Scholastic Competence” in the ‘without XXX’ (e.g., ‘without transitions’ in Table 3) model, too. Otherwise, the comparison between models artificially is inflated by its addition in only the ‘with transitions’ model. Let me put it this way: imagine that transitions did nothing. The ‘with transitions’ model might show better fit (AIC) purely because it adds the pretest of SPPC in the model, right? So I would just put the pretest of SPPC in BOTH models, so that the comparison of models statistics can refer exclusively to the ‘upgrade’ due to transitions. Same comment for Table 4, but substituting ‘mindfulness’ for ‘transitions’. Once again, SPPC pretest should also be in the ‘without mindfulness’ model/column. Same comment for Tables 6-7, and any other table where pretest of SPPC is excluded from the reduced (left) models.

**Response to Comment 1:**

- We appreciate your comment and have made the following changes: in all full models including prior levels of SPPA-SC (school transitions and mindfulness), we have also included prior levels of SPPA-SC to the reduced models for model comparison.  Please see below for our interpretation of this change in the models:
  - For school transitions:
    - Adding prior levels of self-perceived scholastic competence to the reduced model reduced the difference in the AIC value between the reduced and full model. Originally, the model comparison statistics were reported as χ2(3) =34.19, *p*<0.001, AIC difference=28. After adding SPPA-SC into the reduced model, the model comparison statistics are now reported as (χ2(2) = 8.63, *p*<0.001, AIC difference = 5). For the full table, see page 16 in our manuscript. The best fitting model is the full model, including school transitions.
  - For mindfulness:
    - Adding prior levels of self-perceived scholastic competence to the reduced model reduced the difference in the AIC value between the reduced and full model. Originally, the model comparison statistics were reported as χ2(2) =27.33, p<0.001, AIC difference=23.After adding prior levels of SPPA-SC into the reduced model, the model comparison statistics are now reported as χ2(1) = 8.20, *p*=0.004, AIC difference = 6. For the full table, see page 19 in our manuscript. The best fitting model is the full model, including prior levels of trait mindfulness.
  - POST-HOC mindfulness + grades:
    - Adding prior levels of self-perceived scholastic competence to the reduced model reduced the difference in the AIC values between the reduced and full model. Originally, the model comparison statistics were reported as χ2(2) = 30.61, *p*<0.001, AIC difference = 26. After adding prior levels of SPPA-SC to the reduced model, the model comparison statistics are now reported as χ2(1) = 15.32, *p*<0.001, AIC difference = 13. For the full table, see page 22 in our manuscript. The best fitting model is the full model, including prior levels of trait mindfulness.
  - POST-HOC school transitions + grades:
    - Adding prior levels of self-perceived scholastic competence to the reduced model reduced the difference in the AIC value between the reduced and full model. Originally, the model comparison statistics were reported as χ2(3) = 26.21, p<0.001, AIC difference=20. After adding SPPA-SC into the reduced model, the model comparison statistics are now reported as (χ2(2) = 7.20, *p*=0.027, AIC difference = 3). For the full table, see page 23 in our manuscript. The best fitting model is the full model, including school transitions.

**Comment 2:** Add citations for the idea that trait mindfulness is malleable (stated several times on p. 5), rather than just different in different individuals. Was Carlson, 2013 not supposed to be crossed out? If past research does not show this, then make it clearer that this needs to be tested even though it might logically follow from current understandings.

**Response to Comment 2:**

- Thank you for your comment. When referencing the idea of trait mindfulness being malleable, we summarize the research stated in previous sentences. We have added an additional reference to Carlson, 2013 on page 5:
  - “Among adolescents, increasing levels of mindfulness are related to higher academic functioning (Greco et al. 2011) and stronger global self-worth (Brown and Ryan 2003). Expressing and implementing mindfulness in the school-based context is recognized by educators as being associated with fostering attention, resilience, and well-being (Garrison Institute, 2005). These findings, among others discussed below (see Carlson 2013), suggest that mindfulness is a potentially malleable factor related to academic achievement, self-perceptions, and well-being. Due to the malleable nature of trait mindfulness, we propose gaining further understanding of the relationship between trait mindfulness and self-perceived scholastic competence could have implications in improving how individuals feel about themselves in an academic setting. For example, if higher levels of trait mindfulness is found to be related to increased self-perceived scholastic competence than future work could focus on improving trait mindfulness to mimic this effect.”

**Comment 3:** Why the focus on girls? This needs to be addressed more explicitly, though it may not be a big change. Page 4 discusses gender a but, but a sentence stating, “We focus on girls because…” would be helpful.

**Response to Comment 3:**

- We have restructured the below paragraph on pg 4 to make our reasoning for focusing on adolescent girls clearer. See the updated paragraph below.
- Changes made are underlined:
  - “We focus on girls because a study in Portugal found that girls experience a significant decrease in positive academic self-concept following the transition to secondary school (Coelho and Romão 2017). Furthermore, the age range of 10-14 years brings the start of puberty. Puberty is accompanied by a host of transitions, including shifts in values, motivation, and goals related to identity (Pfeifer and Allen 2021; Pfeifer and Berkman 2018). Individuals who have had their first period could be more vulnerable to the negative effect of a school transition (Cantin and Boivin 2004; Juraska and Willing 2017; Simmons et al. 1987). Specifically, Cantin & Boivin (2004) found that individuals who experienced menarche before the transition into middle school had higher rates of negative self-perceptions than individuals who had not experienced menarche. Therefore, further understanding how academic self-perceptions changes over time in girls is important because they may have biological and social vulnerabilities to poorer self-concept during social transitions. Further, distinguishing the impact of the transition into middle school compared to the transition into high school on self-perceived scholastic competence is needed to understand the unique challenges of transitions in hopes of promoting more positive self-perceived scholastic competence during the early adolescent and mid-adolescent period.”

**Comment 4 (originally Comment 14):** Ultimately, this is the editor’s call. But I’m still finding this figure #2 really puzzling. I am not a developmental psychologist and so can appreciate if I’m being dense. Perhaps this is a more common type of figure in longitudinal analyses. However, I simply do not understand what it shows. The revised caption only partially helps.

What do the bars indicate, /exactly/? Saying they “represent the transition” or that they are “the effect” is not helping me understand. Are those means?

I’d once again just dropping the figure, as I don’t see what it adds beyond the tables. This isn’t an interaction - it’s simply first-order “main” effects (I think). Does it even need a figure? Could the main text unpack it, if it very important to communicate something?

Again, apologies that I’m not ‘getting it’ and I am not trying to be difficult. But I do suspect that some readers would share my confusion.

**Response to Comment 4 (originally Comment 14):**

- We apologize for the confusion. We have decided to remove the figure from the manuscript to avoid any potential reader confusion. We believe that the text explaining the results of the analysis is sufficient.

**Comment 5 (originally comment 10):** Two issues here. First, the phrasing about using sums because of the perceived issue with means was not just used for the mindfulness measure, but also for the SPPC measure, but no revision was made for the latter. Second, I would simply recommend DROPPING the language about using sums to avoid means from participants with too little data. I get that there’s another justification (which makes sense: it’s what the scale authors want). But THIS explanation is a red herring and is therefore confusing. I also note Reviewer 2 was confused by this. I think the authors mean the following: “using our software’s SUM function means that participants with missing data on a given scale are listwise deleted for that scale, which we want. We didn’t want to use a MEAN-based function because it would calculate means even for respondents with some missing data, which we see as problematic.” But the thing is, this explanation makes no sense because you can just as easily calculate mean scores that drop participants who didn’t answer X% of items (see the sjstats package for R). So I would really just recommend dropping this extra bit, as it’s confusing.

**Response to Comment 5 (originally Comment 10):**

- Thank you for your comment regarding the language used to justify using sum scores. We have removed the following two sentences from the manuscript as suggested:
- Changes:
  - Pg 11. REMOVED: “We used sum scores rather than mean scores to avoid including participants with mean scores that were derived from too few items.”
  - Pg 12. REMOVED: “We used sum scores rather than mean scores due to accordance with previous validated usage and to avoid including participants with mean scores that were derived from too few items (Greco et al. 2011).”

**Comment 6 (originally Comment 16):** I am good with the response, but just noted this minor detail. Here is the quote: “Another potential pathway could be that receiving better grades leads to increased competence, and we recommend further work to help determine the directionality of these relationships”

I’ve mentioned before, but really all references to ‘competence’ that mean ‘self-perceived competence’ should say the latter. This is particularly problematic because the authors do use actual grades at some points, which could be mistaken as being ‘competence’. Self-perceived competence makes this unambiguous.

**Response to Comment 6 (originally Comment 16):**

- Thank you for finding this error, we have made the following revision and have further examined the manuscript for other confusing uses of the term “competence”:
- Changes:
  - Pg 35. “Another potential pathway could be that receiving better grades leads to an increase in self-perceived scholastic competence, and we recommend further work to help determine the directionality of these relationships.”

**Editor Final Decision:** Accept

Nov 29, 2022

Dear Clare F. McCann,

I have now had a chance to read over your manuscript “Trait mindfulness supports self-perceived scholastic competence in adolescent girls*”, along with the letter describing the changes you made. Thank you for your responsiveness to the concerns that the reviewer and I raised. I am happy to say that your paper is now officially accepted for publication in Collabra: Psychology. Congratulations on this excellent work, I think it will make an important contribution to the literature and I look forward to seeing it published! I hope your experiences with Collabra: Psychology have been positive and that you will continue to consider it as an outlet for your work.

As there are no further reviewer revisions to make, you do not have to complete any tasks at this point.

You will be receiving separate correspondence regarding any production and technical comments, data deposits, as well as publication charges. We work with the Copyright Clearance Center to process any applicable APC charges. Please note that your APC transaction must be completed before your article gets published.

You will have an opportunity to check the page proofs before we publish your article. Thank you again for publishing in Collabra: Psychology.

Sincerely,
Clare Conry-Murray

# Reviewer 1

##### Rating scale questions

|  | Strongly Disagree | Disagree | Neutral | Agree | Strongly Agree |
| --- | --- | --- | --- | --- | --- |
| The study/studies in this manuscript have strong construct validity (good measures and/or manipulations of the constructs the authors wish to study). (Choose “Neutral” if this is not an empirical manuscript) |  |  |  |  | ✔ |
| The study/studies in this manuscript have strong statistical validity (appropriate statistical tests, assumptions are clear and reasonable, no statistical errors, appropriate statistical inferences, etc.). (Choose “Neutral” if this is not an empirical manuscript) |  |  |  | ✔ |  |
| The study/studies in this manuscript have strong internal validity (any causal claims or implications are well-justified, alternative explanations are thoroughly considered, etc.). (Choose “Neutral” if this is not an empirical manuscript, or no causal claims are made or even vaguely implied.) |  |  | ✔ |  |  |
| The study/studies in this manuscript have strong external validity (authors appropriately constrain their conclusions based on the limits of the generalizability of their findings to other contexts (including from lab to real world), other populations, other stimuli or measures, etc.) |  |  |  | ✔ |  |

##### Open response questions

### Please write your review here. The author(s) will see this review. Your identity will not be revealed to the authors unless you also include your name (i.e., sign your review) in this box. It is up to you whether to reveal your identity or not, either is fine.

Apologies for a late review. I have reviewed the list of changes to the manuscript, and the manuscript itself, and I think the authors have done a good job addressing my remaining concerns. I was pleased that the model-level statistics worked out with what I saw as an error removed, as I anticipated they would. The remaining changes I saw as relatively modest, but I do think that the clarity of the manuscript is a bit bolstered by the overall set of changes. I think this is an interesting topic and I wish the authors the best in their future research.
