# Supplementary material for: Trait Mindfulness Supports Self-perceived Scholastic Competence in Adolescent Girls
Source: Collabra Psychol. Author manuscript; Available in PMC 2026 Apr 7. (PMC13052386; doi:10.1525/collabra.57559)
Supplement: Peer Review History [file NIHMS2161622-supplement-Peer_Review_History.docx]

**SUPPLEMENTARY:**

The analyses were repeated without the inclusion of covariates, household income bracket, and age at menarche, to assess for the robustness given the amount of missing data and we wanted to make sure that we were not inadvertently skewing things because of the people that didn’t have specific covariates.

***Mindfulness & Self-perceived Scholastic Competence:***

When examined without covariates, we found that mindfulness had an unstandardized effect size of 0.04 with a p-value of 0.175. With the covariates, mindfulness had an unstandardized effect size of 0.11 with a p-value of 0.008. This 0.07 change in effect size could suggest that the addition of the covariates do matter since the effect size of mindfulness becomes stronger when looking at the model with the covariates.

***School Transitions & Self-perceived Scholastic Competence:***

When we examined the model without household income bracket and menarche, the transition into high school explained significant variance in self-perceived scholastic competence but the transition into middle school did not (χ^2^(3) =59.07, *p*<0.001, AIC difference=53). These results suggest that with the transition into high school, self-perceived scholastic competence score increases on average.

***How much do prior levels of mindfulness additionally predict self-perceived scholastic competence?***

We initially found no relationship between prior levels of mindfulness and self-perceived scholastic competence in the model that did not include covariates (beyond time-lagged self-perceived scholastic competence). We further explored how the inclusion of this time-lagged dependent variable impacted our findings. When not controlling for prior levels of self-perceived self-competence, we found that prior levels of mindfulness explain additional variance in self-perceived scholastic competence scores. In this model, prior levels of self-reported mindfulness predict a 0.07 ± 0.03 increase in SPPA-SC scores (χ^2^(1) =4.56, *p*=0.033; Table 5).

| Table 5. *Post-hoc analyses of self-perceived scholastic competence as predicted by only prior levels of mindfulness* | | |  |
| --- | --- | --- | --- |
|  | *Dependent variable:* | |  |
|  |  | |  |
|  | Self-perceived Scholastic Competence | |  |
|  | Without predictors | Only Mindfulness |  |
|  | (1) | (2) |  |
|  | | |  |
| Prior Levels of Mindfulness |  | 0.070^**^ (0.033) |  |
| Constant | 14.826^***^ (0.322) | 12.976^***^ (0.922) |  |
|  | | |  |
| Observations | 158 | 158 |  |
| Log Likelihood | -414.348 | -412.066 |  |
| Akaike Inf. Crit. | 834.696 | 832.131 |  |
|  |  |  |  |
| *Note:* | | | ^*^p^<0.1; >**^p^<0.05; >***^p<0.01 |
|  |  | |  |

Full Correlation Plot:
